# Supplementary material for: Population dynamics of foxes during restricted-area culling in Britain: Advancing understanding through state-space modelling of culling records
Source: PLoS One. 2019 Nov 19;14(11):e0225201. doi: 10.1371/journal.pone.0225201 (PMC6863561; doi:10.1371/journal.pone.0225201)
Supplement: S1 Appendix — (PDF) [file pone.0225201.s001.pdf]

S1 Appendix. Timing of fox breeding events

Methods

Cubs become vulnerable to detection during lamping when they are weaned and active above ground away from the earth. To account for cub recruitment into the detectable fox population, the time at which this occurs must be defined. In Britain, mating occurs December-February and following a gestation period of 52 days, most births occur between mid-March and mid-April [1]. Female foxes lactate for four weeks following the birth of cubs, and the cubs are fully weaned at 6-8 weeks of age [1]. At about six weeks of age the cubs begin to emerge from the earths, initially only exploring the area around the earth entrance, but become active away from the earth by eight weeks [2]. While most cubs are born in late March, we considered the assumption that all cubs are weaned in one two-weekly time-step and become detectable at the same time was too rigid. A more realistic approach would be to specify a weaned cub distribution incorporating variation around the mean date of recruitment to the detectable fox population to allow for some early or late litters across an estate.

The first step in obtaining this distribution was to use data from fox populations in SE England and Wales describing the probable conception dates of female foxes killed during pregnancy [2]. These data were summarised by taking the mean date and percentage of paired sampling points (*n*=5) on the ascending limb of the curves, i.e. the percentage of females that had conceived over time across both populations). Next, assuming that these mean data points followed a logistic distribution, the 'fitdistr' function from the MASS package [3] in the R statistical software [4] was used to estimate the maximum likelihood values of the parameters describing the cumulative distribution function of the logistic

distribution. This cumulative distribution was taken to describe the distribution of conception events in the female fox population over time.

Given the distribution of conception events it was trivial to project this forward in time, first to a distribution of cub births by assuming a gestation period of 52 days [1], and then to a distribution of cub weaning by assuming that all cubs were weaned and active away from the breeding earth at eight weeks of age (56 days). As expected, the mean of the distribution of births was in late March. This distribution was then used to calculate the proportion of cubs weaned on a given day and thereby vulnerable to lamping in the detectable population. These proportions were then aggregated into a two-weekly time step t for use in the model, with w_t defined as the proportion of weaned cubs entering the detectable population during t (the values of w_t sum to 1).

Results and Conclusions

The estimated distribution of conception events from the data in Lloyd [2] is shown in Fig A, together with the projected distributions of births and weaned cubs. Under these projections, >50% of cubs born on an estate would be recruited to the 'detectable' weaned population during the third and fourth weeks of May, with >80% recruiting during May. These projected distributions are supported by data from 74 estates across Britain on the numbers of cubs killed per week at breeding earths (n=626 cubs, unpublished data, GWCT). This shows that very few cubs were killed before the start of the projected distribution of births (<1%) and that only a small proportion of cubs were killed at earths following the end of the projected distribution of weaned cubs (<5%). We conclude that the projected distribution of weaned cubs provides a suitable description of the timing of when cubs would become active away from breeding earths.

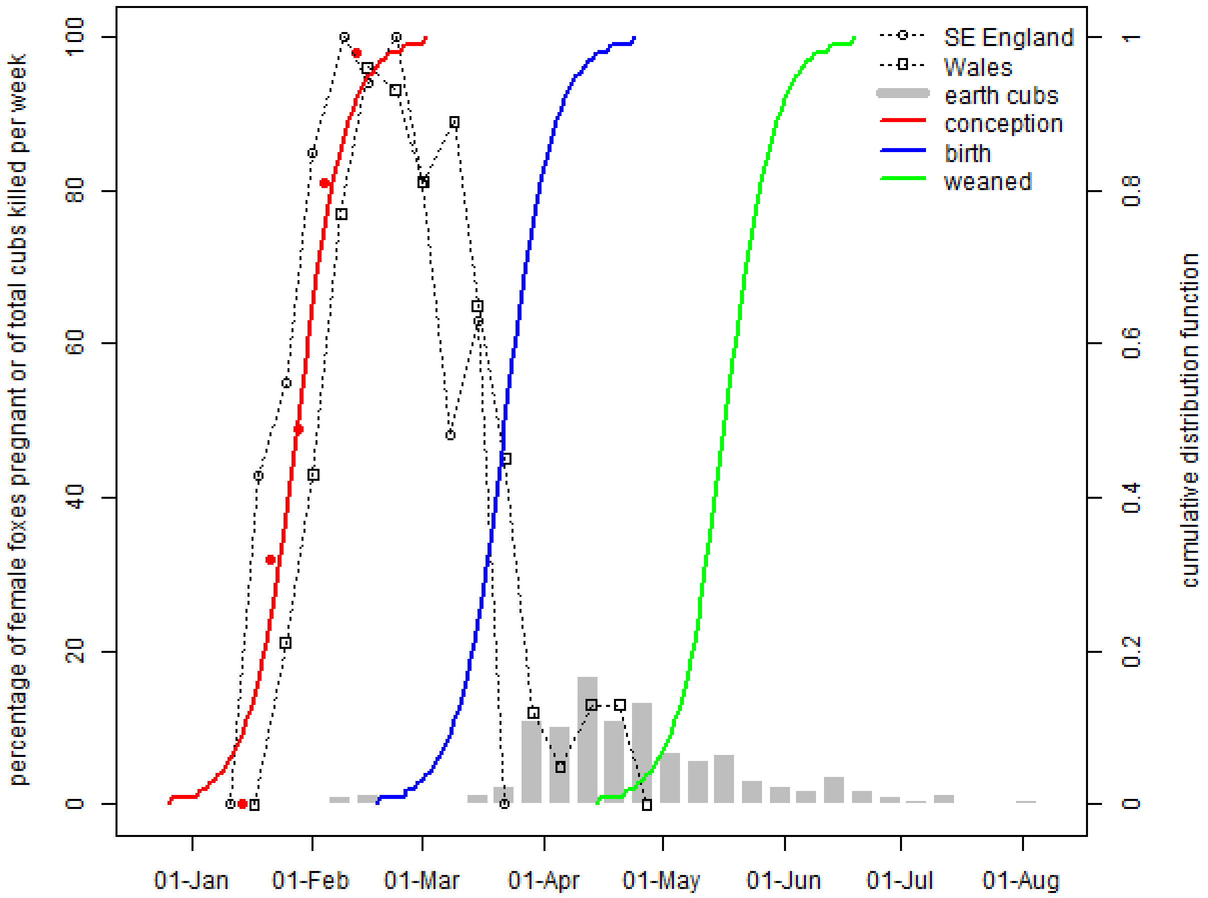

Fig A. Timing of fox breeding events. Data from two British fox populations on the percentage of female foxes that were pregnant at different times during the breeding season [2] are summarised, with mean dates and percentages (red points) used to fit a cumulative logistic distribution to show the percentage of the female population that had conceived over time. The distribution of births was obtained assuming a gestation period of 52 days, from which the distribution of weaned cubs was obtained by assuming that all cubs were weaned at eight weeks of age. The weaned distribution describes when cubs become vulnerable to lamping effort. The histogram shows the percentage of total cubs killed at earths per week on 74 estates across Britain (*n*=626 cubs).

References

1. Macdonald DW, Reynolds JC. Red Fox *Vulpes vulpes*. In: Sillero-Zubiri C, Hoffmann M, Macdonald DW, editors. Canids: Foxes, Wolves, Jackals and Dogs Status Survey and Conservation Action Plan. Gland, Switzerland, and Cambridge, UK: IUCN/SSC Canid Specialist Group; 2004. pp. 129–136.
2. Lloyd HG. The Red Fox. London, UK: Batsford; 1980.
3. Venables WN, Ripley BD. Modern Applied Statistics with S. 4th ed. New York, NY, USA: Springer; 2002.
4. R Core Team. R: A language and environment for statistical computing [Internet]. Vienna, Austria: R Foundation for Statistical Computing; 2018. Available: http://www.R-project.org/
