# Supplementary material for: Population dynamics of foxes during restricted-area culling in Britain: Advancing understanding through state-space modelling of culling records
Source: PLoS One. 2019 Nov 19;14(11):e0225201. doi: 10.1371/journal.pone.0225201 (PMC6863561; doi:10.1371/journal.pone.0225201)
Supplement: S2 Appendix — (PDF) [file pone.0225201.s002.pdf]

S2 Appendix. Choice of process error standard deviation

Most of the earliest applications of state-space models in ecology were in fisheries (e.g. [1,2]), where a value of 0.05 for the standard deviation in process error, σ_p, is considered low and a value of 0.20 for σ_p is considered high for annual time steps [3]. Intuitively, terrestrial systems are subject to higher levels of process variability compared to marine systems because the environment is less stable and human actions cause additional variability. We compiled estimates of σ_p from terrestrial state-space modelling applications (Table A), with estimates larger than 0.20 seemingly common in populations of mammal and bird species (e.g. [4,5]).

These state-space models estimated process errors on annual time-steps because they were modelling recruitment-only and not immigration. Because of the multiplicative nature of lognormal process error, two-weekly values of σ_p must be smaller than for annual time-step models. But modelling immigration as well as recruitment in our model potentially increases the process variation. Our model assumes a constant immigration rate, but realistically the number of foxes moving onto or off the culling area may vary substantially around this rate from one time-step to the next. At the upper extreme, during the post-weaning period, vixens may move with family groups onto or off an estate. This degree of variation would not be allowed if σ_p was fixed at too small a value. An additional reason to expect higher process variation in a restricted-area fox population is relatively low population numbers. Given a density of 2 fox km^{-2} across an estate of 5 km^2, the number of foxes is small enough that loss or gain of a single fox results in a large (10%) proportional change in density, a considerable change highlighting how noisy fox population processes are at this scale.

The most taxonomically relevant estimate of σ_p=0.08 was from a model of the Scandinavian wolf population [6], except that these authors modelled recruitment-only on an annual time-step, and the wolf population considered was >200 individuals. An estimate of σ_p=0.15 on a monthly time-step from an Australian population of the spectacled flying-fox (*Pteropus conspicillatus*) was more temporally relevant, but also came from a recruitment-only model where the population numbered >100,000 individuals [7]. We expected process variation in our fox population data to be higher, so fixed σ_p=0.2 on a two-weekly time step and examined the sensitivity to this choice.

Table A. Mean estimates of annual process error standard deviation σ_p from terrestrial species. Ranges of values indicate estimates obtained either from more than one population or from using alternative estimation methods

Species	σ_p	Reference
Mammals		
Red deer/Elk *Cervus elaphus*	0.0003-0.14	[8]
Reindeer/Caribou *Rangifer tarandus*	0.003-0.25	[8]
Wolf *Canis lupus*	0.08	[6]
Desert bighorn sheep *Ovis canadensis*	0.14-0.21	[9]
Sika deer *Cervus nippon*	0.22	[10]
Red kangaroo *Macropus rufus*	0.28	[4]
Birds		
Eurasian sparrowhawk *Accipiter nisus*	0.07	[11]
Greater snow goose *Chen caerulescens atlantica*	0.10-0.14	[12]
Ovenbird *Seiurus aurocapilla*	0.11-0.18	[13]
Puerto-Rican parrot *Amazona vittata*	0.12	[11]
Canvasback *Aythya valisineria*	0.14	[14]
California condor *Gymnogyps californianus*	0.21-0.30	[11]
Scaly-naped pigeon *Patagioenas squamosa*	0.25	[15]
Greater sage grouse *Centrocercus urophasianus*	0.55	[5]
Greater sage grouse *Centrocercus urophasianus*	0.62	[16]

References

1. Schnute JT. A general framework for developing sequential fisheries models. Can J Fish Aquat Sci. 1994;51: 1676–1688. doi:10.1139/f94-168
2. Meyer R, Millar RB. BUGS in Bayesian stock assessments. Can J Fish Aquat Sci. 1999;56: 1078–1086.
3. Thorson JT, Ono K, Munch SB. A Bayesian approach to identifying and compensating for model misspecification in population models. Ecology. 2014;95: 329–341. doi:10.1890/13-0187.1
4. Knape J, Jonzén N, Sköld M. On observation distributions for state space models of population survey data. J Anim Ecol. 2011;80: 1269–1277. doi:10.1111/j.1365-2656.2011.01868.x
5. Monroe AP, Aldridge CL, Assal TJ, Veblen KE, Pyke DA, Casazza ML. Patterns in Greater Sage-grouse population dynamics correspond with public grazing records at broad scales. Ecol Appl. 2017;27: 1096–1107. doi:10.1002/eap.1512
6. Liberg O, Chapron G, Wabakken P, Pedersen HC, Hobbs NT, Sand H. Shoot, shovel and shut up: cryptic poaching slows restoration of a large carnivore in Europe. Proc R Soc Lond Biol Sci. 2012;279: 910–915. doi:10.1098/rspb.2011.1275
7. Westcott DA, Caley P, Heersink DK, McKeown A. A state-space modelling approach to wildlife monitoring with application to flying-fox abundance. Sci Rep. 2018;8: 4038. doi:10.1038/s41598-018-22294-w
8. Ahrestani FS, Hebblewhite M, Post E. The importance of observation versus process error in analyses of global ungulate populations. Sci Rep. 2013;3: 3125. doi:10.1038/srep03125

9. Colchero F, Medellin RA, Clark JS, Lee R, Katul GG. Predicting population survival under future climate change: density dependence, drought and extraction in an insular bighorn sheep. J Anim Ecol. 2009;78: 666–673. doi:10.1111/j.1365-2656.2009.01528.x
10. Iijima H, Nagaike T, Honda T. Estimation of deer population dynamics using a bayesian state-space model with multiple abundance indices. J Wildl Manag. 2013;77: 1038–1047. doi:10.1002/jwmg.556
11. Hosack GR, Peters GW, Hayes KR. Estimating density dependence and latent population trajectories with unknown observation error. Methods Ecol Evol. 2012;3: 1028–1038. doi:10.1111/j.2041-210X.2012.00218.x
12. Lebreton J-D, Gimenez O. Detecting and estimating density dependence in wildlife populations. J Wildl Manag. 2013;77: 12–23. doi:10.1002/jwmg.425
13. Knape J, Besbeas P, de Valpine P. Using uncertainty estimates in analyses of population time series. Ecology. 2013;94: 2097–2107. doi:10.1890/12-0712.1
14. Gimenez O, Bonner SJ, King R, Parker RA, Brooks SP, Jamieson LE, et al. WinBUGS for population ecologists: Bayesian modeling using Markov Chain Monte Carlo methods. In: Thomas DL, Cooch EG, Conroy MJ, editors. Modeling Demographic Processes in Marked Populations. New York, NY, USA: Springer; 2009. pp. 883–915.
15. Rivera-Milán FF, Boomer GS, Martínez AJ. Monitoring and modeling of population dynamics for the harvest management of scaly-naped pigeons in Puerto Rico. J Wildl Manag. 2014;78: 513–521. doi:10.1002/jwmg.672
16. Ramey II RR, Thorley JL, Ivey AS. Local and population-level responses of Greater sage-grouse to oil and gas development and climatic variation in Wyoming. PeerJ. 2018;6: e5417. doi:10.7717/peerj.5417
