# Supplementary material for: Population dynamics of foxes during restricted-area culling in Britain: Advancing understanding through state-space modelling of culling records
Source: PLoS One. 2019 Nov 19;14(11):e0225201. doi: 10.1371/journal.pone.0225201 (PMC6863561; doi:10.1371/journal.pone.0225201)
Supplement: S3 Appendix — (PDF) [file pone.0225201.s003.pdf]

S3 Appendix. Development of an informative prior for *per capita* birth rate

Methods

There is a biological limit on the number of cubs a female red fox can conceive, so a Bayesian population dynamics model can consider specification of an informative prior probability distribution for the maximum annual *per capita* birth rate *r* that limits the joint-posterior parameter space to biologically realistic values. Assuming that births are density-dependent, maximum *per capita* birth rates should be observed in fox populations that are at very low densities, e.g., those populations suppressed by high culling mortality. This assumption is supported by observations that larger litter sizes occur in regions subject to heavy culling pressure compared to regions with lower culling mortality [1].

Per capita birth rate can be determined from litter size per female data if an assumption is made about the male to female sex ratio in the population. Adult foxes are territorial and the basic social unit is a monogamous pair, although in higher density populations extended social groups are formed in which a dominant pair tolerate several related subordinate females [2–4]. In high density populations the subordinate females are usually non-productive due to high levels of interaction with the dominant female, while in low density populations these females can instead find empty territories in which to disperse into and breed successfully [5]. Populations subject to high levels of culling mortality therefore have a low proportion of non-productive females [6]. From this, a reasonable assumption is that the male to female sex ratio is 50:50, meaning that maximum *per capita* birth rate can be approximated by dividing the litter size per female from a heavily culled population in half. A 50:50 male to female ratio is supported by our fox culling records.

From the 22 estates, eight provided (incomplete) data on the sex of adults killed, and the ratio was 124 males to 109 females, or 53:47.

The maximum litter size reported in the literature is 12 cubs per female [4], but it is not known how intensive the culling was in this population. Use of these data could lead to specification of a uniform prior for *r* that ranges from zero to an upper limit of six fox cubs *per capita*. However, a uniform prior is only vaguely informative as equal weight is given to the values at the extremes of the distribution as to those in the centre. Litter sizes in rural Britain are usually in the range of four to six cubs per female and extreme values (>10 cubs in a litter) are not very common, even in suppressed populations [1,4,7].

A more informative prior probability distribution would show variation around the central tendency of litter size data obtained from heavily culled populations. Heydon & Reynolds [1] studied three largely rural regions of Britain (mid-Wales, east Midlands, East Anglia) and found that fox population density was suppressed by higher culling mortality in two of these regions. Litter sizes were significantly greater in these regions (mid-Wales and East Anglia) compared to a region where culling mortality was lower (east Midlands). The mean and variance of litter sizes from the populations with heavy culling were used to parameterise an informative prior probability distribution for maximum *per capita* birth rate.

All estimated litter sizes per female were determined from counts of dark placental scars from females killed during the March to July period [8]. Litter size estimates were not adjusted for non-productive females because none were found in the heavily culled samples from the mid-Wales and East Anglia [1], supporting the assumption of a 50:50 sex ratio. The mean annual litter sizes per female were 6.38 cubs ± 0.41 in mid-Wales, and 6.24 cubs ± 0.35 in East Anglia (±s.e.), from regional samples of 21 and 45 female foxes, respectively [1]. The standard deviations in sampled litter sizes were calculated from the standard error of the

means using these sample sizes, giving values of 1.88 cubs (mid-Wales) and 2.35 cubs (East Anglia).

As r can take non-integer values it should be characterised by a continuous distribution, so although females can only have discrete litter sizes, the litter size data were assumed to be drawn from a gamma distribution to allow later conversion to *per capita* litter sizes following an assumption of a 50:50 male to female sex ratio. Also, given that the variances in litter size samples were less than the means, the discrete Poisson or negative binomial distributions are unsuitable. The shape, c, and rate, λ, parameters of the gamma distribution were calculated using the mean and variance in litter size samples from each region [9]. These regional gamma distributions were used to generate 500 random numbers to give simulated litter size data that had the same mean and variance as the regional samples. Next, assuming a 50:50 sex ratio, values from the two simulated datasets were pooled across regions and then divided by two to give a gamma-distributed *per capita* litter size dataset.

The maximum likelihood estimates (MLE) of the gamma distribution parameters were obtained from the simulated *per capita* litter size data using the 'fitdistr' function in the MASS package [10] of the R statistical software [11]. The maximum likelihood estimates were almost identical to the analytically calculated values, but using a numerical approach to estimation allowed the standard errors of the estimates to be obtained from the information matrix. The fitted gamma distribution was tested for goodness-of-fit to the simulated *per capita* litter size data using the Kolmogorov-Smirnov test.

Results and Conclusions

The shape parameters of the gamma distributions used to simulate data on regional litter size at birth per female were calculated as 11.53 (mid-Wales) and 7.06 (East Anglia), with the rate parameters calculated as 1.81 (mid-Wales) and 1.13 (East Anglia). The

simulated regional distributions of litter size are shown in Fig A. The *per capita* litter size dataset obtained from these simulated data (Fig B) had a mean of 3.17 cub fox^{-1}yr^{-1} ± 1.07 (s.d.). The CV of this distribution was 0.338.

The gamma distribution fitted to the *per capita* litter size data had a shape parameter MLE of 8.77 ± 12.20 (s.d.) and a rate parameter MLE of 2.76 ± 3.95 (s.d.). These estimates were equal to those obtained by analytically calculating them from the mean and variance of the data (Fig B). As the *P*-value from the Kolmogorov-Smirnov goodness-of-fit test was >0.05 the gamma distribution was determined to be suitable for these data.

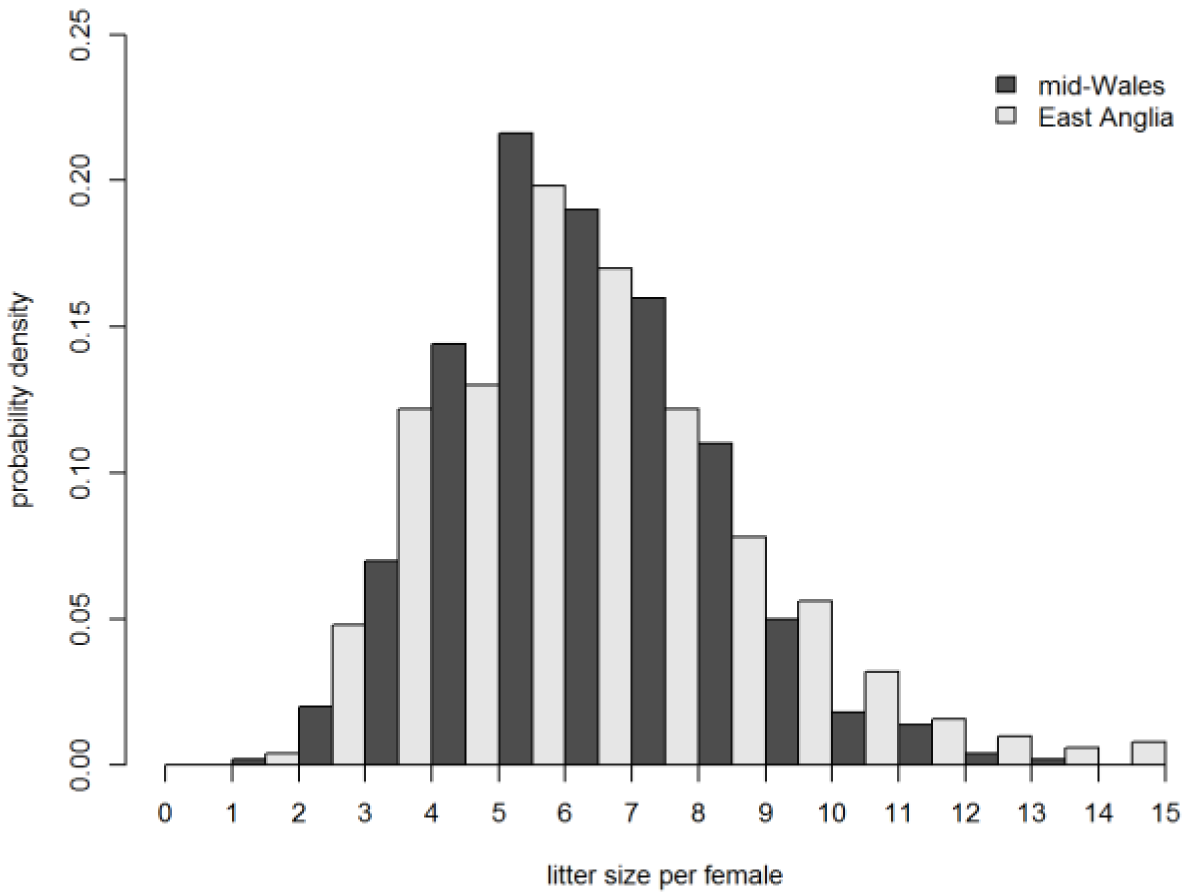

Fig A. Simulated litter size per female data from mid-Wales and East Anglia. Values obtained by generating random numbers from a gamma distribution parameterised using the sample mean and standard deviation in litter size from each region. Data are shown binned into integer values.

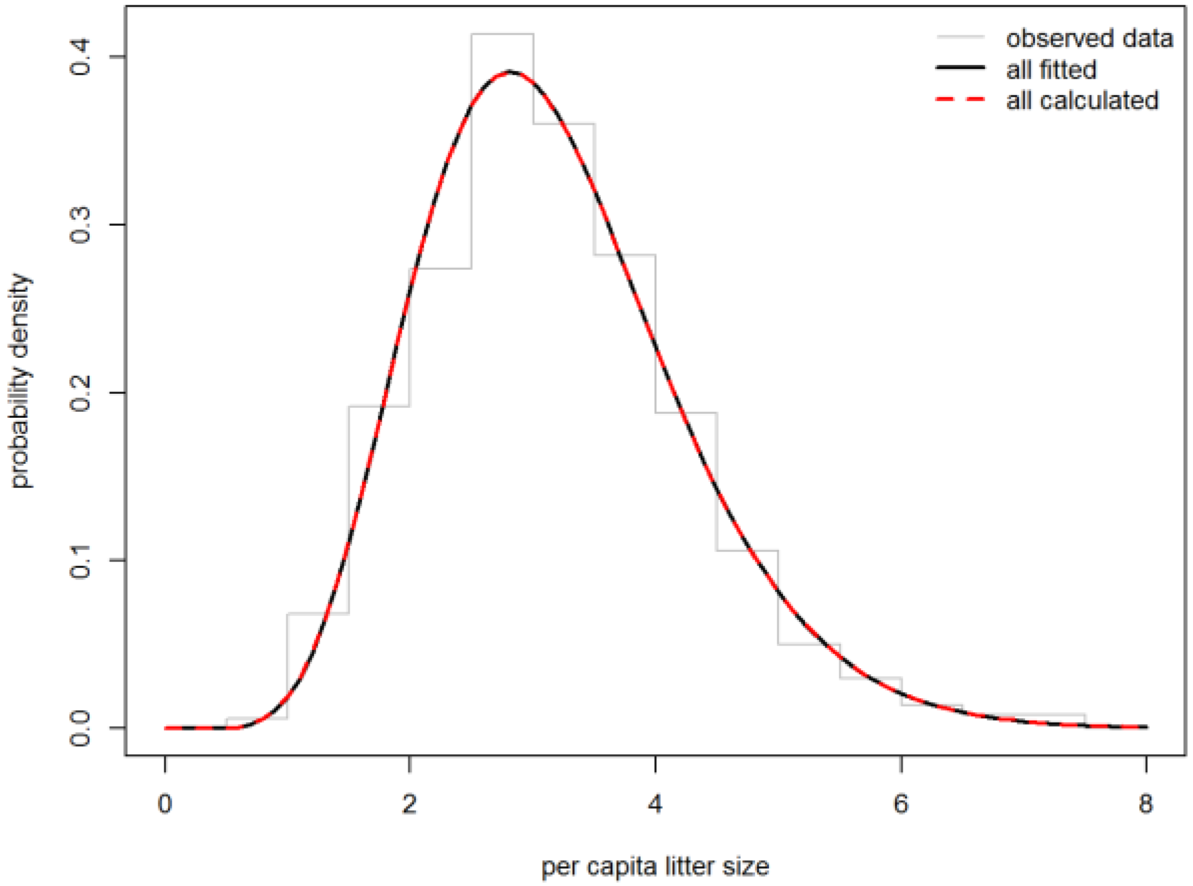

Fig B. Distribution of 'observed' *per capita* birth rate. Gamma probability density functions fitted to these data by maximum likelihood and by analytical calculation of the shape and rate parameters are shown for comparison.

References

1. Heydon MJ, Reynolds JC. Demography of rural foxes (*Vulpes vulpes*) in relation to cull intensity in three contrasting regions of Britain. J Zool. 2000;251: 265–276.
2. Macdonald DW. 'Helpers' in fox society. Nature. 1979;282: 69–71. doi:10.1038/282069a0
3. Reynolds JC, Tapper SC. The ecology of the red fox *Vulpes vulpes* in relation to small game in rural southern England. Wildl Biol. 1995;1: 105–119.
4. Macdonald DW, Reynolds JC. Red Fox *Vulpes vulpes*. In: Sillero-Zubiri C, Hoffmann M, Macdonald DW, editors. Canids: Foxes, Wolves, Jackals and Dogs Status Survey and Conservation Action Plan. Gland, Switzerland, and Cambridge, UK: IUCN/SSC Canid Specialist Group; 2004. pp. 129–136.
5. Macdonald DW. Social factors affecting reproduction amongst red foxes (*Vulpes vulpes* L., 1758). In: Zimen E, editor. The Red Fox: Symposium on Behaviour and Ecology. The Hague, Netherlands: Dr. W. Junk bv Publishers; 1980. pp. 123–175.
6. Lloyd HG. The Red Fox. London, UK: Batsford; 1980.
7. Voigt DR, Macdonald DW. Variation in the spatial and social behaviour of the red fox, *Vulpes vulpes*. Acta Zool Fenn. 1984;171: 261–265.
8. Lindström ER. Reliability of placental scar counts in the red fox (*Vulpes vulpes* L.) with special reference to fading of the scars. Mammal Rev. 1981;11: 137–149.
9. Evans M, Hastings NAJ, Peacock JB. Statistical distributions. 3rd ed. New York, NY, USA: Wiley; 2000.
10. Venables WN, Ripley BD. Modern Applied Statistics with S. 4th ed. New York, NY, USA: Springer; 2002.

11. R Core Team. R: A language and environment for statistical computing [Internet]. Vienna, Austria: R Foundation for Statistical Computing; 2018. Available: http://www.R-project.org/
