# Supplementary material for: Population dynamics of foxes during restricted-area culling in Britain: Advancing understanding through state-space modelling of culling records
Source: PLoS One. 2019 Nov 19;14(11):e0225201. doi: 10.1371/journal.pone.0225201 (PMC6863561; doi:10.1371/journal.pone.0225201)
Supplement: S4 Appendix — (PDF) [file pone.0225201.s004.pdf]

S4 Appendix. Simulation-estimation analysis

Methods

Estimation performance of the generalised depletion model within a Bayesian state-space modelling framework was evaluated using simulation-estimation analysis. This consisted of separate steps, detailed below.

Generation of lamping effort data

Lamping effort data were generated to drive detection and culling data simulation. We used the timescale our data for a reference time series length (i.e. January 1996 to August 2000) and generated data on a weekly time step. To produce realistic data, we used data from all 74 gamekeepered estates to characterise seasonal variability in lamping effort. This showed more effort was used in the spring and autumn and less effort in summer and winter, with significant differences between mean weekly effort at the commencement of harvest in mid-July and around Christmas (Fig A). To allow for between-week variation in effort within this annual pattern, but ensure that expected annual effort remained constant, hours of lamping effort per week E_t values were generated as Poisson-distributed random variables with expectation equal to the mean weekly effort values.

We performed two simulation-estimation analyses which aimed to understand: 1) choice between vague or informative priors, and 2) effect of time series length when using informative priors. For analysis one, we obtained the reference E_t time series by multiplying the mean weekly effort values for one year by the length of the reference time series, with effort kept constant across years. We examined the effect of variability in annual culling effort between years, but differences in density and parameter estimate bias were minimal (results not reported

here). For analysis two, we varied the number of breeding seasons covered by the time series to give time series that were two, three, four and five years long. The mean weekly effort values were the same in each year.

Simulation of data

The known ('true') detectable fox population dynamics were simulated using an operating model with known parameters, process errors, and observation errors [1,2]. The operating model contained Eq 2 – Eq 5 (see main Methods), which were used to simulate fox density N_t time series using parameter values defined in Table A.

The operating model also contained a set of equations to simulate the culling data needed to calculate N_t and numbers of fox detections for fitting the model. Weekly cull data were simulated given the assumption that as detectable foxes, they were active away from the earth and vulnerable to both lamping and snaring culling risks (we ignored risks from other culling methods). Given these risks are not equal, the order by which lamping cull L_t or snaring cull S_t occurred in a given week was randomised by simulating a Bernoulli random variable, h_t, that took a value of zero if lamping occurred first, or a value of one if snaring occurred first. Numbers of detections Y_t in a given week were then simulated from N_t as a Poisson-distributed random variable given a weekly lamping effort schedule E_t:

$$Y_t \sim \begin{cases} Pois(dE_tN_t) & h_t = 0 \\ Pois(dE_t[N_t - S_t]) & h_t = 1 \end{cases} \qquad \text{(S1)}$$

where d is the rate of successful search (Table A). L_t was then calculated as:

$$L_t = p_kY_t/A \qquad \text{(S2)}$$

where p_k is the probability of a detected fox being killed (lamping success) and A is the estate area. Estate area was set equal to 10 km^2. The value of p_k was set at 0.30, the mean observed value from our data, but there was large variation in this value reflecting times when

gamekeepers were unable to shoot any of the foxes detected, and others when all foxes detected were shot. This led to the observed distribution of p_k being U-shaped, as most values were either at 0 or 1. To model variation in the probability of lamping success per week, values for p_k were randomly generated from a distribution based upon the beta-binomial. The beta-binomial distribution is a binomial distribution with parameters for the number of trials and probability of success, but whose probability of success is not a constant but it is generated from a beta distribution. This allows U-shaped distributions, but without modification the generated numbers range from zero to the number of trials. To constrain the generated numbers to the [0, 1] interval required, it was necessary to divide them by the number of trials (set equal to 100 here). Numbers were generated using the rbetabinom function from the R package VGAM [3] with values of 0.3 and 0.75 for the 'prob' and 'rho' arguments, respectively. S_t was simulated as:

$$S_t = \begin{cases} p_s(N_t - L_t) & h_t = 0 \\ p_s N_t & h_t = 1 \end{cases} \tag{S3}$$

where p_s is the probability of snaring success per fox per week, which was set equal to 0.05 and assumed to be constant over time. C_t was assumed to be removed immediately prior to recruitment of cubs into N_t in week t:

$$C_t = p_c w_t r N_t (1 - N_t/K) \tag{S4}$$

where p_c is the probability of being killed at an earth per cub, which was set equal to 0.2 and assumed to be constant over time. Data from 39 earths suggest that nearly all (88%) of cubs seen at earths are killed [4]. If the assumption is made that once an earth has been found all cubs are killed, e.g. by using terriers to bolt them above ground to waiting shotguns, this probability will equal the probability of locating an earth on an estate.

A number of initial simulation runs were used to check that the true parameter values (Table A) and lamping effort schedules simulated plausible culling data and fox density time

series that were within the range of known regional (> 1000 km^2) spring and autumn fox densities [5]. Particular attention was given to the value of σ_p such that reasonable week-to-week variation in fox density due to process error was produced (range of variation in N_t was by a factor of 0.7-1.5). For each lamping effort schedule within each part of the simulation-estimation analysis, the operating model was used to simulate 20 datasets to allow the performance of the estimation method to be evaluated.

The base case true parameter values for v, r, M and d in Table A were close to the informative prior medians for these parameters. The sensitivity of the estimation results to the true parameter values used to simulate N_t was examined by simulating two alternative sets of 20 datasets. The true values used in these alternative simulations were: 1) 0.5 times the base case values in Table A for N_0, v, r and M, and 1.5 times the base case value for d, giving fox densities relative to K that were lower than those using the base case set of true values; and 2) 1.5 times the base case values for N_0, v, r and M, and 0.5 times the base case value for d, giving fox densities relative to K that were higher than those using the base set of true values.

Bayesian estimation

The simulated time series of lamping effort, numbers of foxes detected and culled were considered as data and Bayesian estimation was performed on each dataset within the state-space modelling framework to estimate the true fox density and parameter values. The estimation model used was the same as the operating model used to simulate the data, meaning that any differences between simulated and estimated values were due to the performance of the estimation method and not to mis-specification of the model. Samples from the joint posterior probability distribution of the unknown parameters and latent states $p(N_0, K, v, r, M, d, \sigma_p, \boldsymbol{N_t} \mid$ data) were simulated by MCMC integration using WinBUGS 1.4 [6] implemented from within

the R statistical software [7] using the R2WinBUGS package [8]. As in the main Methods, to improve the slow mixing of the Gibbs sampler the population process model equations (Eq 2 – Eq 4) were re-parameterised by expressing fox density as a proportion of carrying capacity ($P_t = N_t / K$, Eq 8 – Eq 10). Eq 11 was thus used in the observation model (instead of Eq 6). We obtained the marginal posterior estimates of N_t as the product of P_t and K.

The joint posterior was estimated from two independent MCMC chains run in parallel with initial values chosen randomly from the joint prior. As in the main study Methods, to conserve computer memory, only 1 in 100 iterations of the Markov chains were recorded after the first 100,000 iterations were removed as the burn-in, and we derived inferences from a sample of 20,000 iterations from two chains of 10,000 iterations. We assessed convergence using the Gelman-Rubin convergence statistic [9].

Measurement of estimation bias

The performance of the estimation method was evaluated by determining how accurately the marginal posterior probability distribution of each parameter and latent state estimated the true value used in the simulation of each dataset. For each parameter θ this was measured using the median of the marginal posterior to compare to the true value and summarised by calculation of the percent relative bias (PRB):

$$\text{PRB} = 100[(\text{estimated } \theta - \text{simulated } \theta)/\text{simulated } \theta] \quad \text{(S5)}$$

Bias plots were used to summarise the PRB for [N_0, K, v, r, M, d, σ_p] across the 20 simulated datasets. The marginal posteriors were also evaluated visually against the true parameter values to examine the precision of parameter estimates.

The PRB in each latent state estimate was similarly calculated by comparing the median of the marginal posterior in each week to the true fox density. The mean of these density PRB

values was then calculated across the length of the time series and summarised across the 20 simulated datasets using bias plots. The median of the marginal posterior for each latent state was also compared visually to the true fox density in each week to examine the fit of the model along the time series.

Results and Conclusions

Influence of prior distribution specification

Estimation models using either vague or informative priors generally resulted in reliable estimates of weekly N_t, as the posterior medians of N_t closely tracked trajectories of simulated 'true' fox density (Fig B). Vague prior model estimates of N_t had wider credible intervals than informative prior model estimates. Use of vague priors did result in mild overestimation of N_t for certain datasets, especially during the summer months; these simulated datasets were characterised by having fewer weeks in which there was lamping effort during the summer. Examination of the mean bias in posterior median N_t estimates across the length of the time series showed that estimation bias of N_t using vague priors was generally low (median PRB <10%), with only a slight bias reduction achieved from using informative priors (median PRB 3%; Fig C). The model using informative priors was the 'best' choice as determined by the Deviance Information Criterion (DIC) in 85% of datasets, with a mean difference of 3.2. Though this difference is only weak evidence in favour of informative priors, the largest differences in DIC were for those simulated datasets where the posterior median fox density using vague priors showed the largest departure from the true values, and so is where the choice is most important.

The influence of informative priors was more apparent when examining the bias in parameter estimates. Despite reliable N_t estimates, the model had identifiability problems when

using vague priors, resulting in estimation bias for some parameters. These findings are similar to those reported elsewhere [10]. During the initial stages of model formulation, maximum likelihood was used to determine whether it was possible to estimate the parameters of an observation-error only model, and most simulated datasets resulted in reasonable estimates and positive-definite Hessian matrices [11]. The non-identifiability was therefore determined to be due to extrinsic redundancy as it was an issue only for some datasets; the use of informative priors overcame this problem.

M was grossly overestimated using the vague prior model, with median PRB almost 200% larger than the true value (Fig C). However, it should be noted that this bias only refers to the central tendency of the marginal posterior, and uncertainty in these estimates of *M* was also large (Fig D). Indeed, the true value of *M* was found within the 80% credible interval (CI) of the marginal posterior for all but one dataset despite the posterior median being an overestimate for all. Strong pairwise posterior correlations between parameter combinations can indicate confounding [12]. Given the poor estimates of *M*, it was expected that *M* would be highly confounded with the other parameters; however, posterior correlations between any of the parameters were not strong (correlation coefficient > 0.7). There were moderate posterior correlations (0.4-0.7) between *M* and *v* (positive) and between *M* and σ_p (positive) in the results from a number of the datasets, but the coefficients between *M* and other parameters were all <0.4, reflecting weak posterior correlation. The use of an informative prior on *M* greatly reduced the bias in estimates of *M* (Fig C) and also had the effect of removing the moderate correlations between *M* and *v* or σ_p.

The vague prior model resulted in precise estimates of both *v* and *d* (Fig D), but *v* was precisely overestimated while *d* was minimally-biased (Fig C). The small positive bias in *v*

estimates likely arose from the positive posterior correlation with *M*. These results were improved with the use of informative priors, which resulted in minimally-biased posterior medians and 80% CIs which encompassed the true value of *v* for all datasets. Both N_0 and *K* also tended to be overestimated when using the vague prior model, with median PRB for both around 40% (Fig C). This was improved to 15% when informative priors were used on the other parameters as the joint posterior space was constrained. However, similar to *M*, the large uncertainty in estimates of *K*, and to a lesser extent of N_0, meant that the true value of N_0 and *K* was found within the 80% CI for all datasets (Fig D).

State-space models with density-dependence are known to have extrinsic redundancy problems [13] as the parameter determining the strength of density-dependence often proves difficult to estimate, especially when observation errors are large [14]. As the CV of observation error was about 20 times larger than the CV of the process error (1.37:0.07) this partly explains why estimates of *K* were biased using both vague and informative prior models. The bias in *K* was higher in those simulated populations where N_t spent little time near to *K*. Culled fox populations can be assumed to be at low densities relative to carrying capacity due to the continual removal of foxes, and these populations were simulated such that N_t would spend most time below 50% of *K*. As a consequence, estimates of *K* were uncertain as N_t did not spend long enough around *K* for density-dependent effects on cub recruitment and immigration to be clearly observed. The estimates of N_t were not greatly affected by the uncertainty in *K* because when N_t is considerably smaller than *K*, the dynamics of the fox population are fairly insensitive to the value of *K* (Fig E). As shown by these simulations, the value of *K* only becomes important if N_0 is relatively large. When N_0 is <50% of *K* there is little difference in N_t trajectories to values of *K* between 4 and 25 fox/km^2.

The true value of *r* was found within the 80% CI of the marginal posteriors under both models (Fig D), but although the small positive bias was similar using both (Fig C), the vague prior model resulted in some median values for *r* >50% different from true values. These estimates corresponded to those datasets which had fewer weeks in which there was lamping effort during the summer months. As a result, it appears insufficient information in the data during the summer months led to overestimation of cub recruitment without an informative prior on *r*, and consequently the overestimation of N_t in these datasets. The extreme overestimates were not found when an informative prior on *r* was used.

Although the bias in σ_p was not large using either vague or informative priors (Fig C), there were some datasets where the true value was outside the 80% CI of the marginal posterior when either type of prior was used (Fig D). This indicated that σ_p was only weakly identifiable. The addition of process error within the state-space modelling framework seemingly influenced the ability of the model to estimate *M* due to the positive posterior correlation between *M* and σ_p. When σ_p was fixed, the positive bias in estimates of *M* using the vague prior model was greatly reduced. In the absence of a suitable informative prior for σ_p, these findings led to the decision to fix σ_p in the application of the model to our data.

Aside from moderate positive posterior correlations between *v* and *M* and between σ_p and *M* from most datasets, there was moderate negative posterior correlation between *r* and *K* in the results from roughly half of them. There was also some moderate negative correlation between *v* and *r* in the results from a couple of datasets, indicating features which made it difficult for the model to determine whether immigration was large and cub production small, or *vice versa*. The most noticeable feature of these particular simulations was the higher number of weeks in which

there was zero lamping effort during the May-June period when weaned cubs were recruited into the population, so there was insufficient information in the data to disentangle these processes.

Effect of time series length

The estimation model was unable to recover N_t trajectories reliably or estimate parameters without bias from shorter time series of <2 years, as although median PRB in mean weekly fox density estimates was close to zero, there were some datasets which resulted in extremely biased results where mean weekly fox density was overestimated by almost 100% (Fig F). Despite these time series being around 100 weekly time steps long, they only covered two cub recruitment periods. This appears to provide insufficient information to separately estimate v and r, as shown by the negative posterior correlation between these parameters when time series were short. This highlights a further challenge to using state-space models to estimate density-dependent terms as parameters may be strongly correlated if there are not enough informative time steps in the data [15]. Estimation was improved as time series became longer and more cub recruitment periods were covered, and N_t trajectories were generally reliably estimated for time series that were $\geq$3 years long.

The length of time series did have a large effect on the ability to reliably estimate certain parameters, indicating identifiability issues when fewer time steps were present in the data. In particular, time series of $\leq$2 years resulted in large overestimates of K and σ_p, with median PRB of 65% and 30%, respectively (Fig Fa). Examination of the marginal posteriors for these parameters showed that the bias in K resulted from minimal posterior updates to the prior, with the bias reflecting where the true value for K (4.0 fox km^{-2}) was in relation to the median of the prior (6.9 fox km^{-2}). For σ_p the marginal posteriors were hugely variable, which unlike for K meant that the true value was often outside of the 80% CI for σ_p. These biases were reduced as

time series became longer (Fig Fb-d). For any length of time series, there was minimal bias bin estimates of *v*, *r*, *M* and *d* (Fig F), but in the shorter time series this was largely due to the influence of the informative priors, which, especially for *r* and *M*, received minimal posterior updates.

There were no strong posterior correlations between any of the parameters estimated using datasets of any length, and the use of informative priors meant that even with short time series, moderate posterior correlations were limited to one or two parameter pairs per dataset. Across the results from all datasets, the mean number of parameter pairs showing moderate correlation decreased as the time series became longer, from 1.3 (range 0-3) in 2-year time series to 0.9 (range 0-1) in 5-year time series, showing there was clearly more correlation when time series were shorter. In shorter time series, posterior correlation was most commonly found between N_0 and σ_p (positive) and between *v* and *r* (negative); while in longer time series negative correlation between *r* and *K* was more common.

In addition to overall time series length, estimates were affected by how many weeks' lamping effort there were in each year. The ability of the gamekeeper to keep the fox population at a low density is dependent upon how much culling effort is used, meaning that culling success and reliable estimation of the population dynamics are interlinked. Lamping is only one of a suite of culling methods available and, as our data show, there is a seasonal variation in when it is used. This means the detection rate data can be quite sparse during certain times of year, and several weeks (or months) can pass without any lamping effort being used. Weekly estimates of N_t from periods without lamping effort were often poor because without detection rate data to fit the model to it became increasingly difficult to distinguish between observation and process errors. In addition, sufficient weeks with lamping effort are needed during the May-June cub

recruitment period to be able to estimate *r*, regardless of whether an informative prior is used. From these results, we recommend a detection rate time series must contain a minimum of eight weeks of lamping effort per year, with no gap in the time series longer than nine months, and with ≥2 weeks of lamping effort during the cub recruitment period. Time series with little lamping effort in the first year resulted in greater N_0 estimation bias, while high effort levels during the first year resulted in minimal bias. Accordingly, consistent lamping effort during the first three months was determined to be a requirement for reliable estimation of N_0.

Sensitivity to true parameter values

The sensitivity of results to the values used for N_0, *v*, *r*, *M* and *d* to simulate the weekly N_t trajectories showed that when compared to the base case set of true values, the use of true values that were low or high compared to the informative prior medians did affect the estimations when these priors were used in the estimation model. However, the effect on the ability of the model to recover the true fox density was small as the posterior medians of N_t tracked the true fox density well when N_t relative to *K* was either lower (Fig G) or higher (Fig H) than the base case. Across the length of the time series, the mean percent relative bias in posterior median N_t from using lower or higher true values was 9% and 11%, respectively, compared to 3% with the base case values.

The sensitivity of parameter estimates to alternative sets of true values was generally increased when the simulated fox density was lower than under the base case values. In the low-density population case the worst estimates were of *M*, which was consistently overestimated relative to the lower true value in this case. The median PRB was 119% and true values were below the 80% CI of the marginal posteriors for four datasets (Fig Ia, Table B). Given the minimal posterior updating of the informative prior for *M*, this further suggested that *M* may only

be weakly identifiable using this model when σ_p is also estimated. Nevertheless, sensitivity of the reconstructed N_t trajectories to estimation bias in M was low.

r was also overestimated in the low-density population case, with a median PRB of 47% and true values below the 80% CI for six datasets (Fig Ia, Table B). This was expected as the lower value in this case (1.4 cub fox^{-1}yr^{-1}) was only in the second percentile of the prior distribution for *r*, so given this extreme true value the data would need to be very informative to override the prior central tendency. Although the posteriors showed that the prior was updated downwards in all datasets, this resulted in the consistent overestimation of *r*. In contrast, *K* was better estimated than under base case values as median PRB indicated that estimates of *K* were minimally-biased (Fig Ia, Table B). While *d* was minimally-biased when base case values were used, *d* was underestimated relative to the higher true value in this case, although median estimates were on average within 12% of true values (Fig Ia, Table B). One reason why *d* might not be estimated as well in a low-density population could be that fewer foxes leads to more zero detection events and therefore less information with which the model can separate true from false zeros. The median estimates for other parameters were all within 5% of the base case (Fig Ia, Table B). The marginal posteriors for σ_p were overestimated on average by 9%, but this was not consistent between datasets and for many the true values were close to either the upper or lower 80% CI (two were above, one below). Apart from *r* and *M*, the true values for the other parameters were within the 80% CI for nearly all (>85%) datasets.

In the high-density population case, *r* and *M* were typically underestimated relative to their higher true values, although there was more posterior updating for these parameters than seen in the low-density population case (Fig Ib). This meant the bias was less, with median PRB being only –9% (*r*) and –16% (*M*). True values of *r* were outside the 80% CI of the marginal

posteriors for only two datasets, and true values for *M* were within all 80% CIs. This case did result in poorer estimates of *K* as median estimates were on average 38% higher than the true value, although the true value for *K* was outside of the 80% CI of the marginal posterior for only two datasets. This highlighted a feature of the alternative simulation cases, as convergence on the joint posterior was not as straightforward as under the base case set of values, with the worst bias seen with those datasets for which convergence was most difficult. This was especially the situation for *K* in the high-density population case. The median estimates for other parameters in this case were on average all within 5% of the base case, with true values within the 80% CI for nearly all datasets. Posterior correlations in the results using the alternative sets of true values were similar to those observed under the base case set of values when using informative priors.

Tables

Table A. Parameter definitions, values used to simulate data under different conditions (base case, low density population, high density population), and prior probability distributions used for each estimated model parameter in simulation-estimation analysis.

**Parameter**	**Symbol**	**Units**	**Value for simulation**			**Vague prior**	**Informative prior**
			**Base**	**Low**	**High**		
Initial density	N_0	fox km^{-2}	2.0	1.0	3.0	~ uniform(0.001, 13.9)	not specified
Carrying capacity	K	fox km^{-2}	4.0	4.0	4.0	~ uniform(0.001, 13.9)	not specified
Immigration rate	v	fox km^{-2} wk^{-1}	0.046	0.023	0.069	~ uniform(0, 1)	~ lognormal(μ = -3.079, σ = 0.8)
Per capita birth rate	r	cub fox^{-1} yr^{-1}	2.8	1.4	4.2	~ uniform(0, 6)	~ gamma(c = 8.77, λ = 2.76)
Instantaneous non-culling mortality rate	M	wk^{-1}	0.009	0.0045	0.0135	~ uniform(0, 0.1)	~ lognormal(μ = -4.711, σ = 0.6)
Rate of successful search	d	km^{2} hr^{-1}	2.0	3.0	1.0	~ uniform(0, 10)	~ lognormal(μ = 0.693, σ = 0.6)
Process error standard deviation	σ_p	-	0.15	0.15	0.15	~ uniform(0.001, 0.5)	not specified
Estate area	A	km^{2}	10	10	10	n/a	n/a
P(detected fox being killed)	p_k	-	0.3	0.3	0.3	n/a	n/a
P(snaring success per fox per week)	p_s	-	0.05	0.05	0.05	n/a	n/a
P(being killed at earth per cub)	p_c	-	0.2	0.2	0.2	n/a	n/a

Table B. Mean Percent Relative Bias in the medians of the marginal posterior probability distributions from 20 populations relative to different sets of true parameter values used to simulate them: the base set of values in Table A and two alternative sets of values; one giving a low fox density population (0.5[N_0, v, r, M], 1.5[d]) and one giving a high fox population (1.5[N_0, v, r, M], 0.5[d]). K and σ_p were unchanged from the base case. All estimation models used the same informative priors on v, r, M and d.

**Parameter**	**Base case (%)**	**Low density (%)**	**High density (%)**
N_0	15.2	19.2	14.8
K	14.9	1.3	38.0
v	3.3	3.6	3.6
r	9.4	46.9	-9.3
M	8.9	118.7	-15.5
d	-0.3	-12.3	-4.9
σ_p	6.4	8.6	-2.3
$\widetilde{N_t}$	3.3	9.2	11.1

Figures

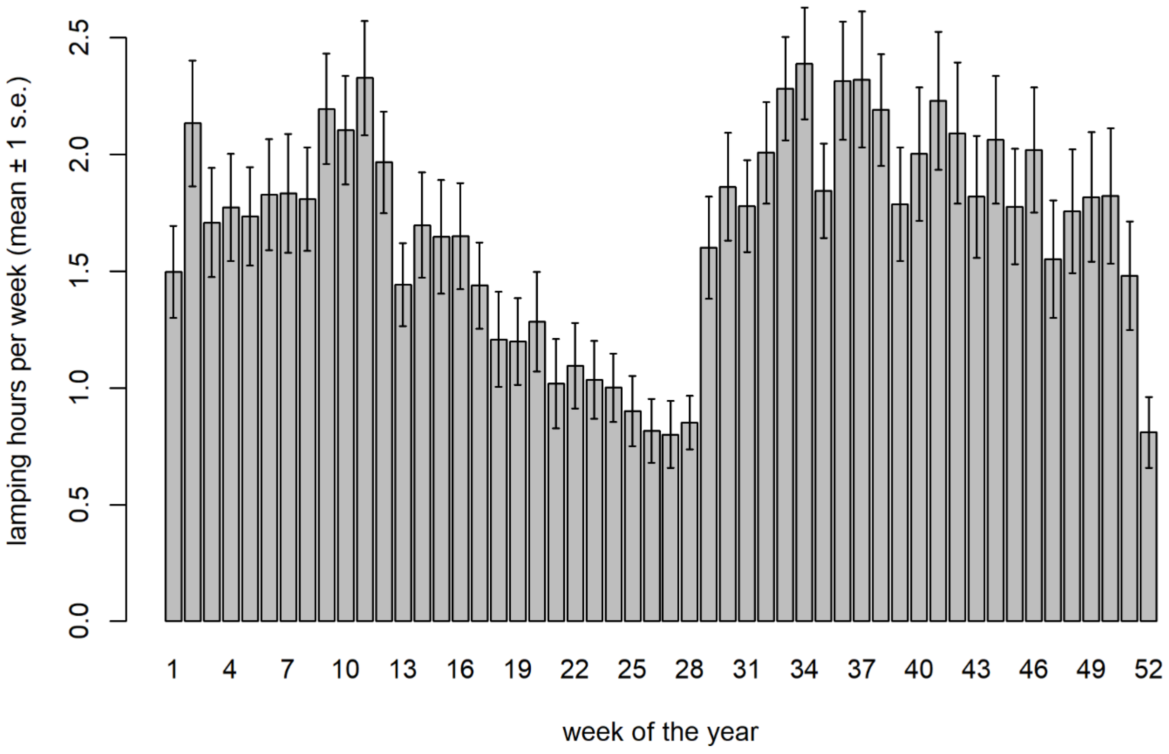

Fig A. Mean hours of lamping effort used per week throughout the year across a sample of 74 gamekeepered estates from the Fox Monitoring Scheme (unpublished data, GWCT).

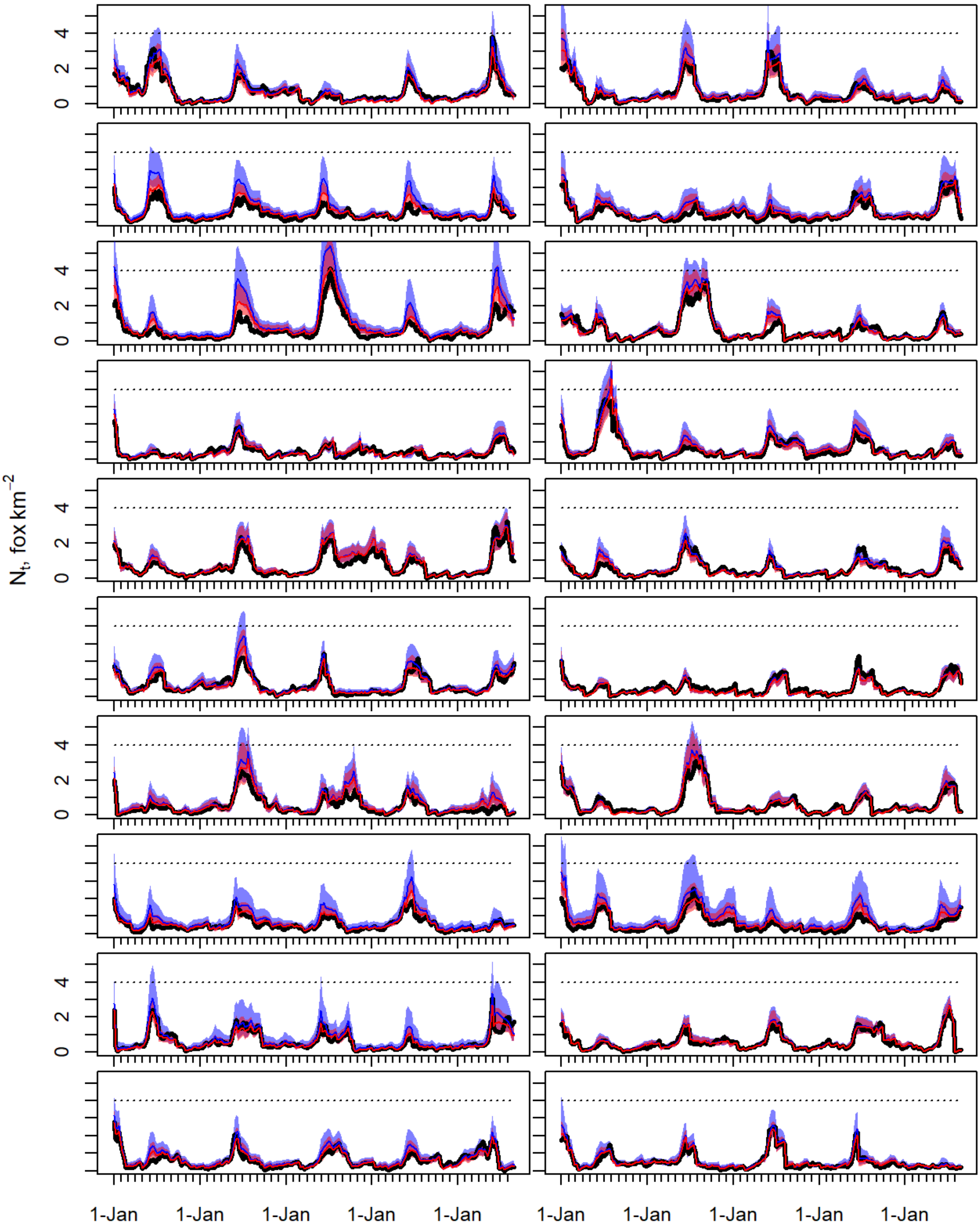

Fig B. Fox density time series from 20 culled populations simulated on a weekly time step (black). The medians of the posterior probability distributions for weekly fox density estimated using an estimation model with either a) vague priors (blue) on all model parameters, or b) informative priors (red) on *v*, *r*, *M* and *d*, with vague priors on the other parameters, are plotted. Blue and red shading shows the 80% credible intervals for vague prior and informative prior models, respectively. Dotted line shows true value of *K*.

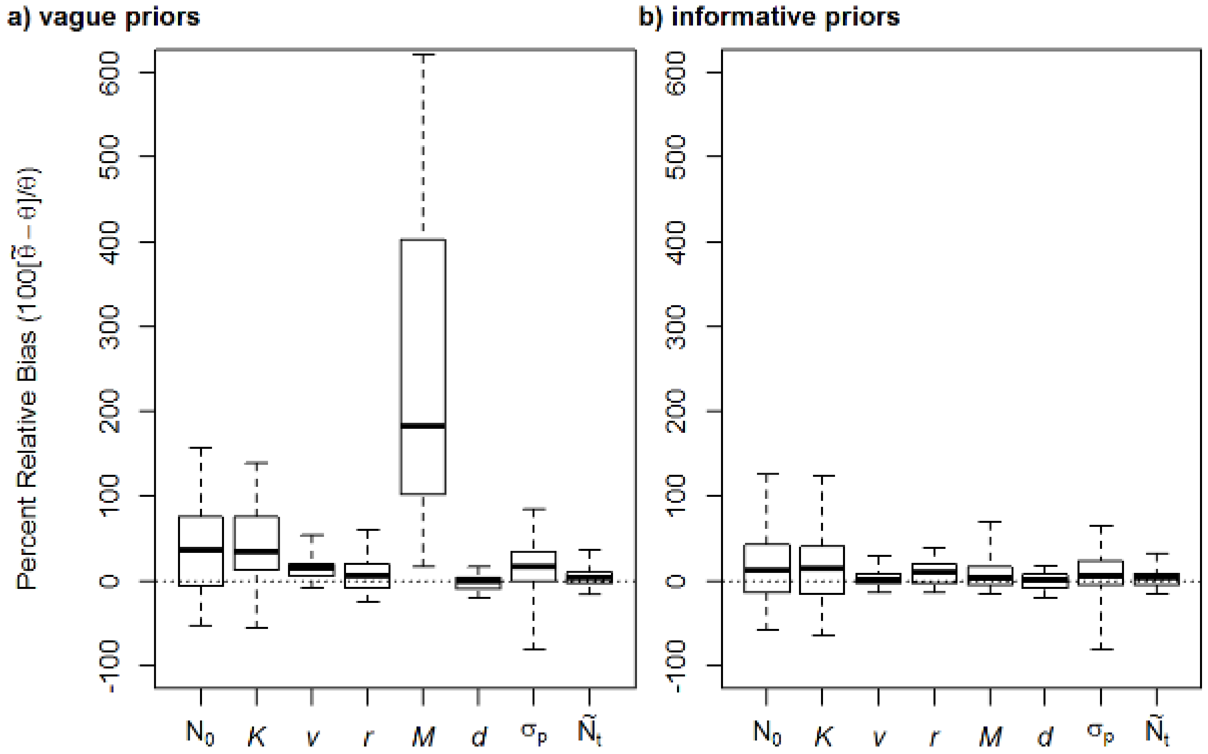

Fig C. Distributions of percent relative bias (PRB) in the median of the marginal posterior probability distributions from 20 culled populations relative to the true parameter values used to simulate the populations on a weekly time step. In addition to the PRB in parameter estimates, the mean PRB in weekly N_t is shown. The estimation model used either a) vague priors on all model parameters, or b) informative priors on *v*, *r*, *M* and *d*, with vague priors on the other parameters. The median PRB for each parameter is shown as a black bar, boxes represent the interquartile range (IQR), and whiskers represent the range of PRB.

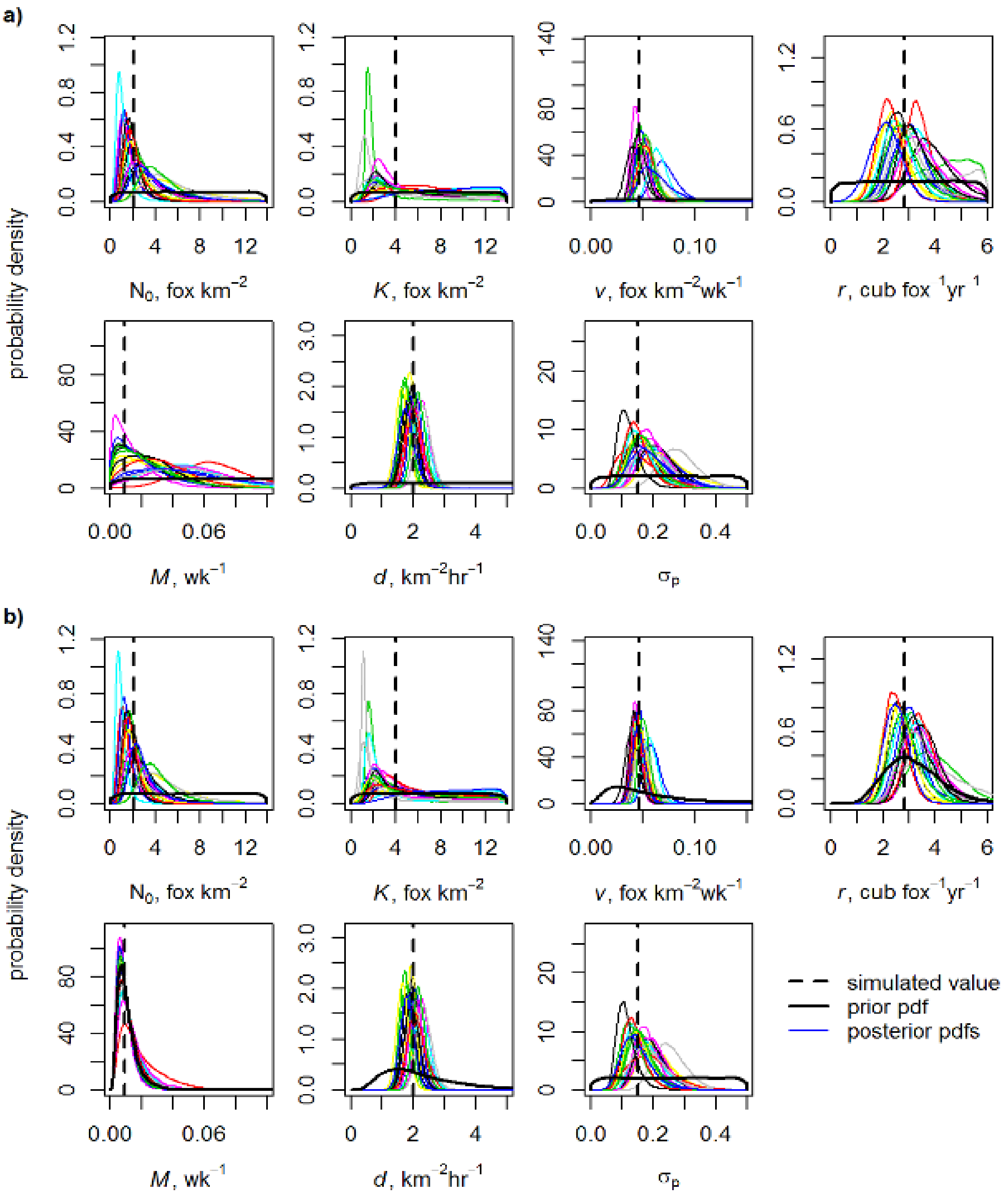

Fig D. Profiles of the marginal posterior probability distributions of parameters estimated from 20 culled populations simulated on a weekly time step. The estimation model used either a) vague priors on all model parameters or b) informative priors on *v*, *r*, *M* and *d*, with vague priors on the other parameters. Each coloured line represents one simulation; solid black line represents the prior, vertical dashed line represents the true parameter value used in the simulations.

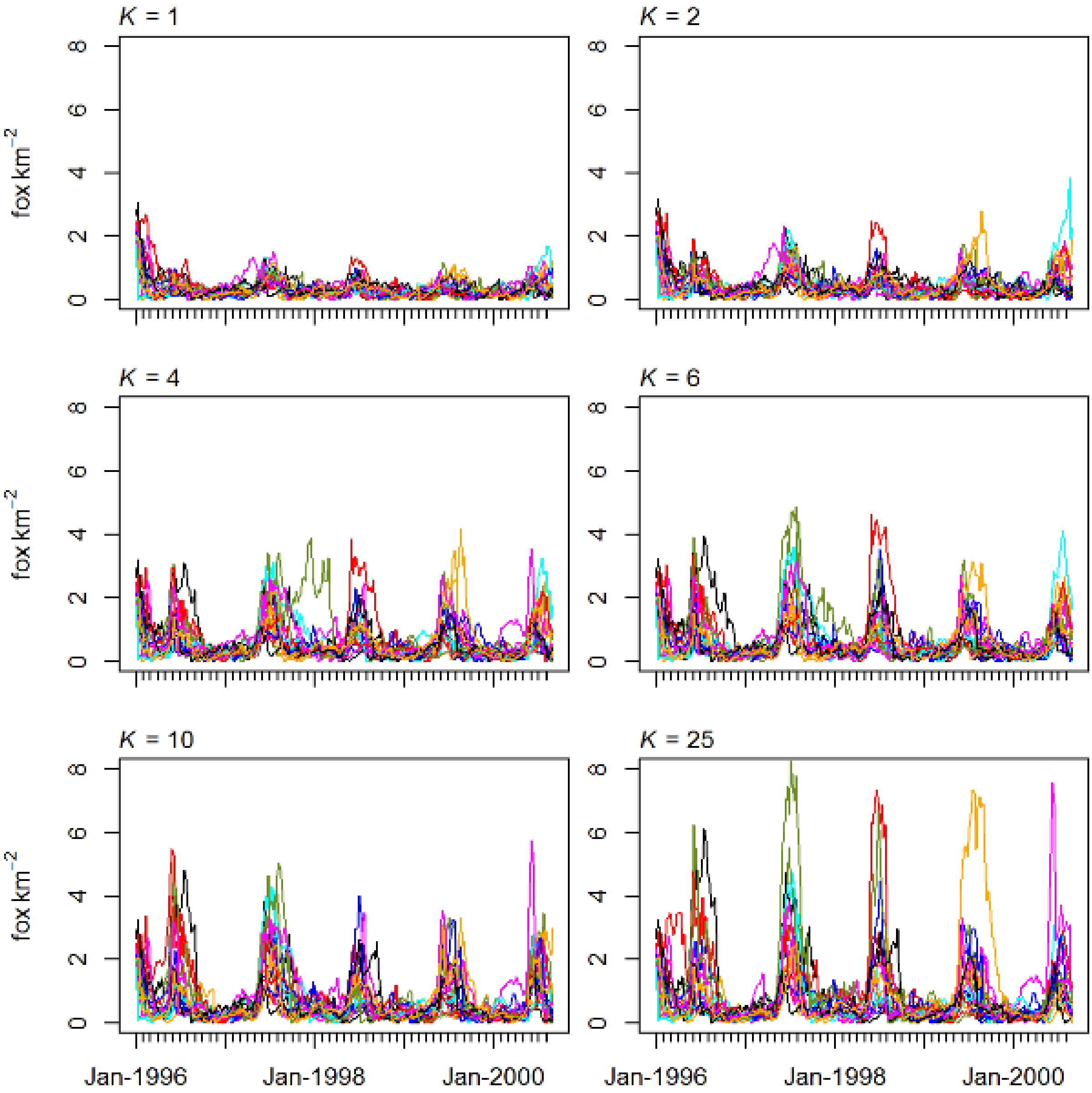

Fig E. Simulated fox density of 20 culled populations under different carrying capacity values. All other parameter values were as defined in Table A (reference case is K=4 fox km^{-2}).

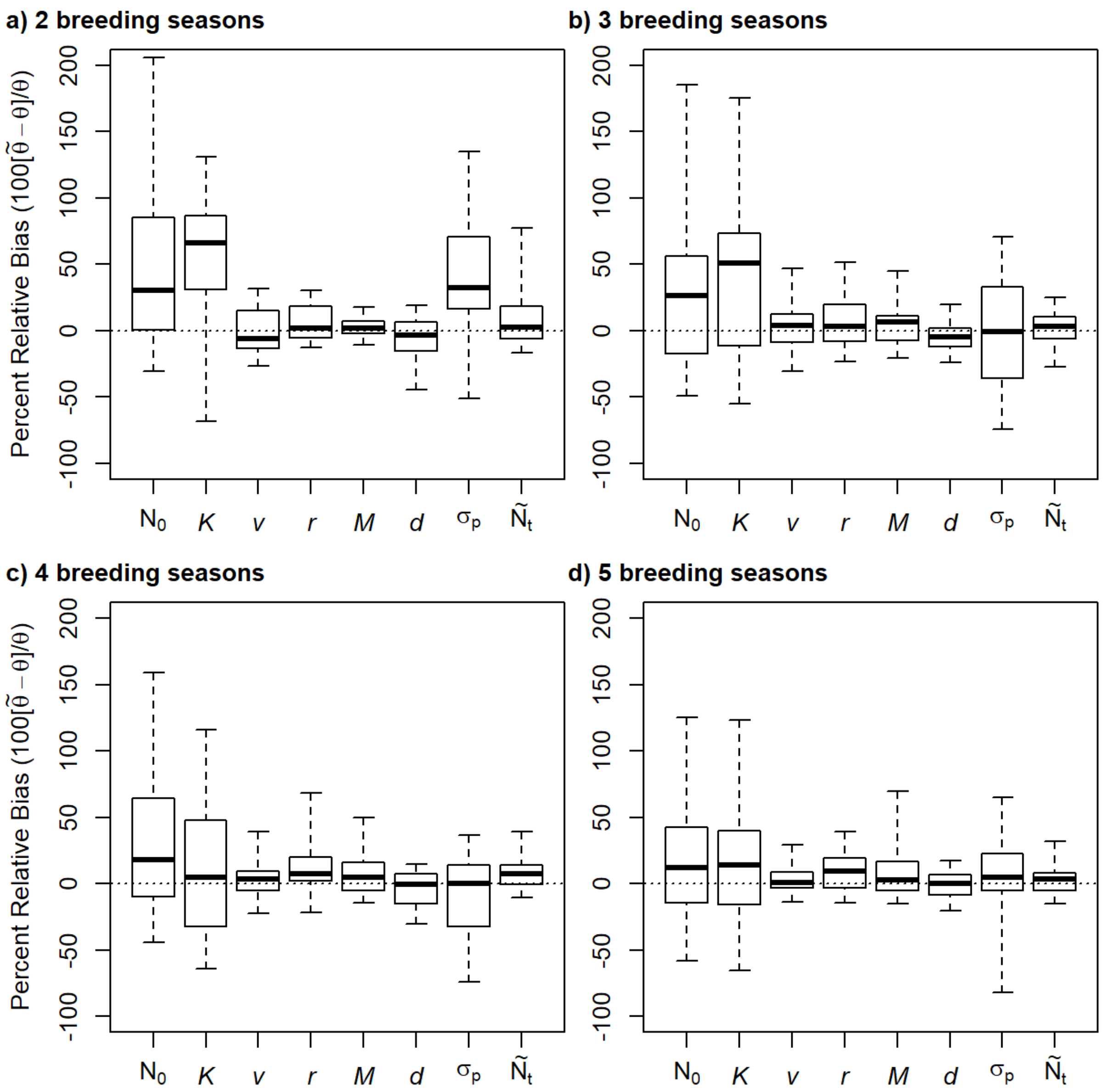

Fig F. Distributions of percent relative bias (PRB) in the median of the marginal posterior probability distributions from 20 culled populations relative to the true parameter values used to simulate the populations on a weekly time step. In addition to the PRB in parameter estimates, the mean PRB in weekly N_t is shown. Differences in PRB due to detection rate time series that covered a) two, b) three, c) four, or d) five breeding seasons are shown. The estimation model used informative priors on *v*, *r*, *M* and *d*, with vague priors on the other parameters. The median PRB for each parameter is shown as a black bar, boxes represent the interquartile range (IQR), and whiskers represent the range of PRB.

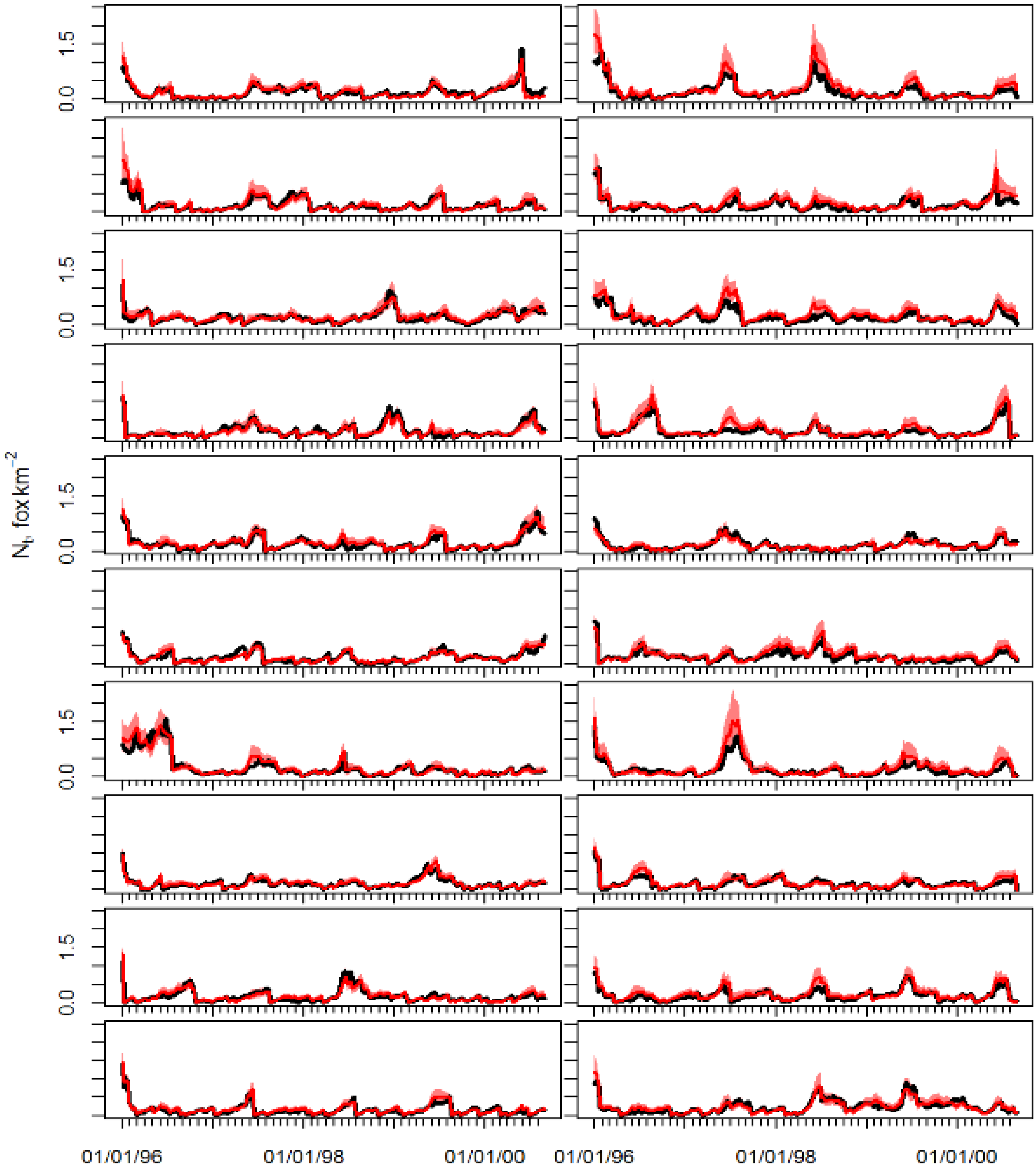

Fig G. Fox density time series from 20 culled populations simulated on a weekly time step (black). Data were simulated using true values 0.5 times the base case values for N_0, *v*, *r* and *M*, and 1.5 times the base case value for *d* in Table A, giving lower fox densities relative to *K* (4 fox km^{-2}) than the reference set in Fig B. The medians of the posterior probability distributions for weekly fox density estimated using an estimation model with informative priors (red) on *v*, *r*, *M* and *d*, with vague priors on the other parameters are plotted. Red shading shows the 80% credible interval.

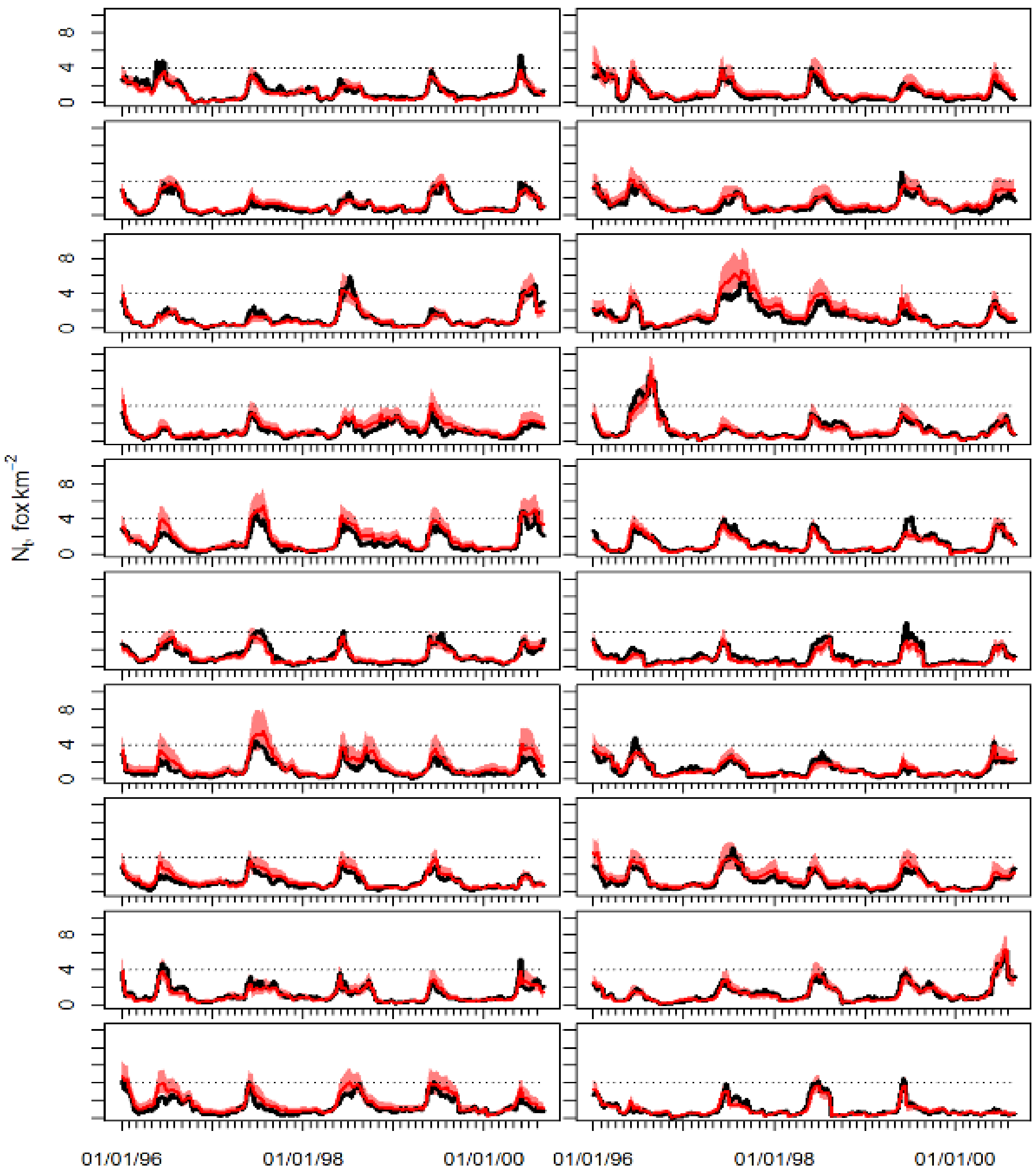

Fig H. Fox density time series from 20 culled populations simulated on a weekly time step (black). Data were simulated using true values 1.5 times the base case values for N_0, *v*, *r* and *M*, and 0.5 times the base case value for *d* in Table A, giving higher fox densities relative to *K* (4 fox km^{-2}) than the reference set in Fig B. The medians of the posterior probability distributions for weekly fox density estimated using an estimation model with informative priors (red) on *v*, *r*, *M* and *d*, with vague priors on the other parameters are plotted. Red shading shows the 80% credible interval. Dotted line shows true value of *K*.

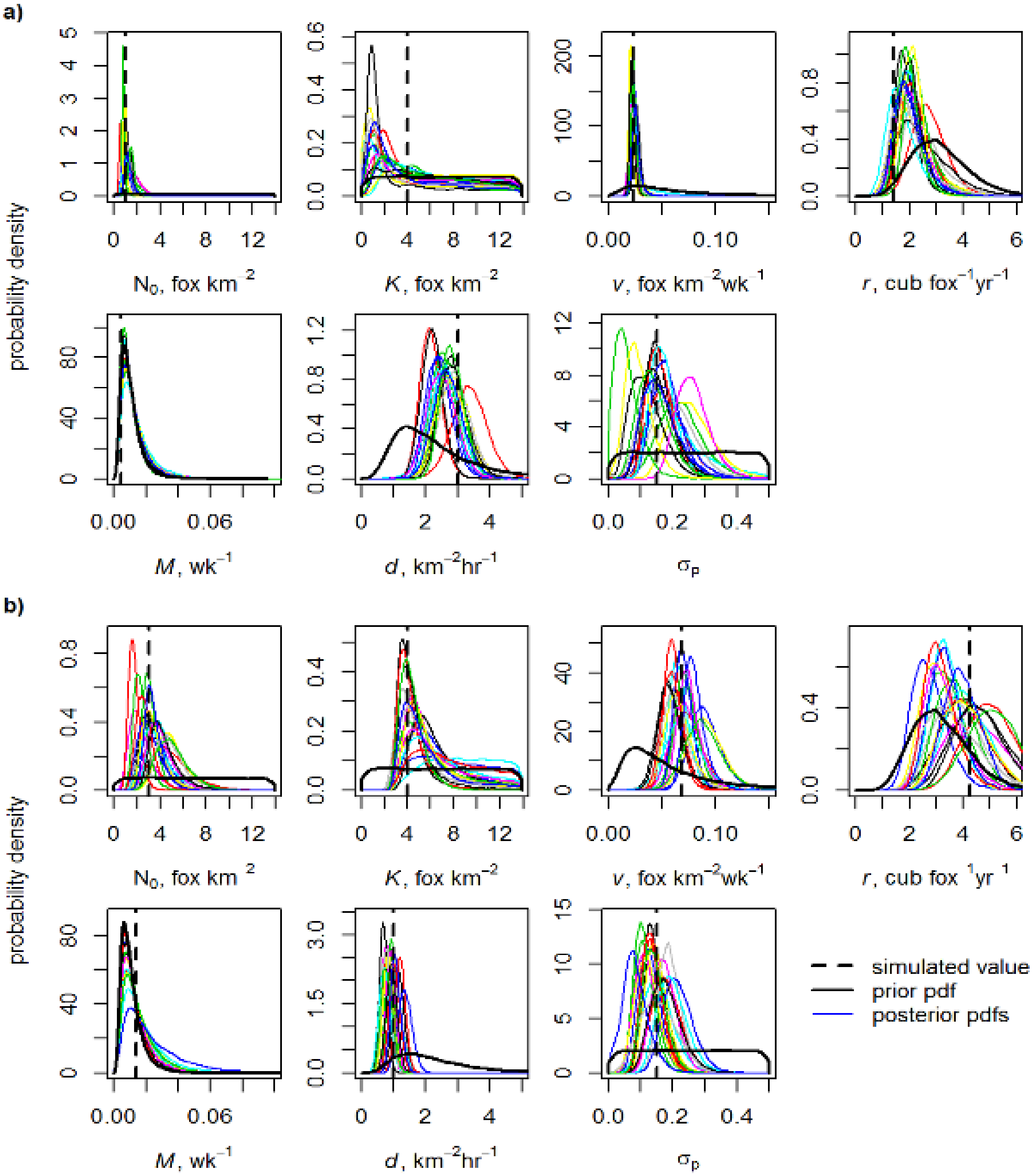

Fig I. Profiles of the marginal posterior probability distributions of parameters estimated from 20 culled populations simulated on a weekly time step. Data were simulated using true values a) 0.5 times the base case values for N_0, *v*, *r* and *M*, and 1.5 times the base case value for *d* in Table A, or b) 1.5 times the base case values for N_0, *v*, *r* and *M*, and 0.5 times the base case value for *d*. The estimation model used informative priors on *v*, *r*, *M* and *d*, with vague priors on the other parameters. Each coloured line represents one simulation; the solid black line represents the prior, vertical dashed line represents the true parameter value used in the simulations (to be compared to posterior median).

References

1. McAllister MK, Starr PJ, Restrepo VR, Kirkwood GP. Formulating quantitative methods to evaluate fishery-management systems: what fishery processes should be modelled and what trade-offs should be made? ICES J Mar Sci. 1999;56: 900–916.
2. Rademeyer RA, Plagányi ÉE, Butterworth DS. Tips and tricks in designing management procedures. ICES J Mar Sci J Cons. 2007;64: 618–625. doi:10.1093/icesjms/fsm050
3. Yee TW. The VGAM Package for Categorical Data Analysis. J Stat Softw. 2010;32: 1–34.
4. Reynolds JC. Fox control in the countryside. Fordingbridge, UK: The Game Conservancy Trust; 2000.
5. Heydon MJ, Reynolds JC, Short MJ. Variation in abundance of foxes (*Vulpes vulpes*) between three regions of rural Britain, in relation to landscape and other variables. J Zool. 2000;251: 253–264.
6. Spiegelhalter DJ, Thomas A, Best NG, Lunn DJ. WinBUGS [Internet]. Cambridge, UK: Medical Research Council Biostatistics Unit; 2007. Available: https://www.mrc-bsu.cam.ac.uk/software/bugs/the-bugs-project-winbugs/
7. R Core Team. R: A language and environment for statistical computing [Internet]. Vienna, Austria: R Foundation for Statistical Computing; 2018. Available: http://www.R-project.org/
8. Sturtz S, Ligges U, Gelman A. R2WinBUGS: a package for running WinBUGS from R. J Stat Softw. 2005;12: 1–16.
9. Gelman A, Carlin JB, Stern HS, Rubin DB. Bayesian Data Analysis. 2nd ed. London, UK: Chapman & Hall; 2004.

10. Dunham K, Grand JB. Effects of model complexity and priors on estimation using sequential importance sampling/resampling for species conservation. Ecol Model. 2016;340: 28–36. doi:10.1016/j.ecolmodel.2016.08.010
11. Gimenez O, Viallefont A, Catchpole EA, Choquet R, Morgan BJT. Methods for investigating parameter redundancy. Anim Biodivers Conserv. 2004;27: 561–572.
12. Trenkel VM. A two-stage biomass random effects model for stock assessment without catches: What can be estimated using only biomass survey indices? Can J Fish Aquat Sci. 2008;65: 1024–1035. doi:10.1139/F08-028
13. Kéry M, Schaub M. State-Space Models for Population Counts. In: Kéry M, Schaub M, editors. Bayesian Population Analysis using WinBUGS: A Hierarchical Perspective. Boston, MA, USA: Academic Press; 2012. pp. 115–132.
14. Knape J. Estimability of density dependence in models of time series data. Ecology. 2008;89: 2994–3000. doi:10.1890/08-0071.1
15. Lebreton J-D, Gimenez O. Detecting and estimating density dependence in wildlife populations. J Wildl Manag. 2013;77: 12–23. doi:10.1002/jwmg.425
