# Supplementary material for: Population dynamics of foxes during restricted-area culling in Britain: Advancing understanding through state-space modelling of culling records
Source: PLoS One. 2019 Nov 19;14(11):e0225201. doi: 10.1371/journal.pone.0225201 (PMC6863561; doi:10.1371/journal.pone.0225201)
Supplement: S5 Appendix — (PDF) [file pone.0225201.s005.pdf]

S5 Appendix. Fox density and parameter estimates from modelled estates

This Appendix contains the posterior outputs from modelled estates not included in the main Results section (i.e. all apart from DLQ). Five estates used as case studies together with DLQ (main Results, Fig 3) are shown first (Figs A-E), followed by other estates in alphabetical order by estate code (Figs F-U).

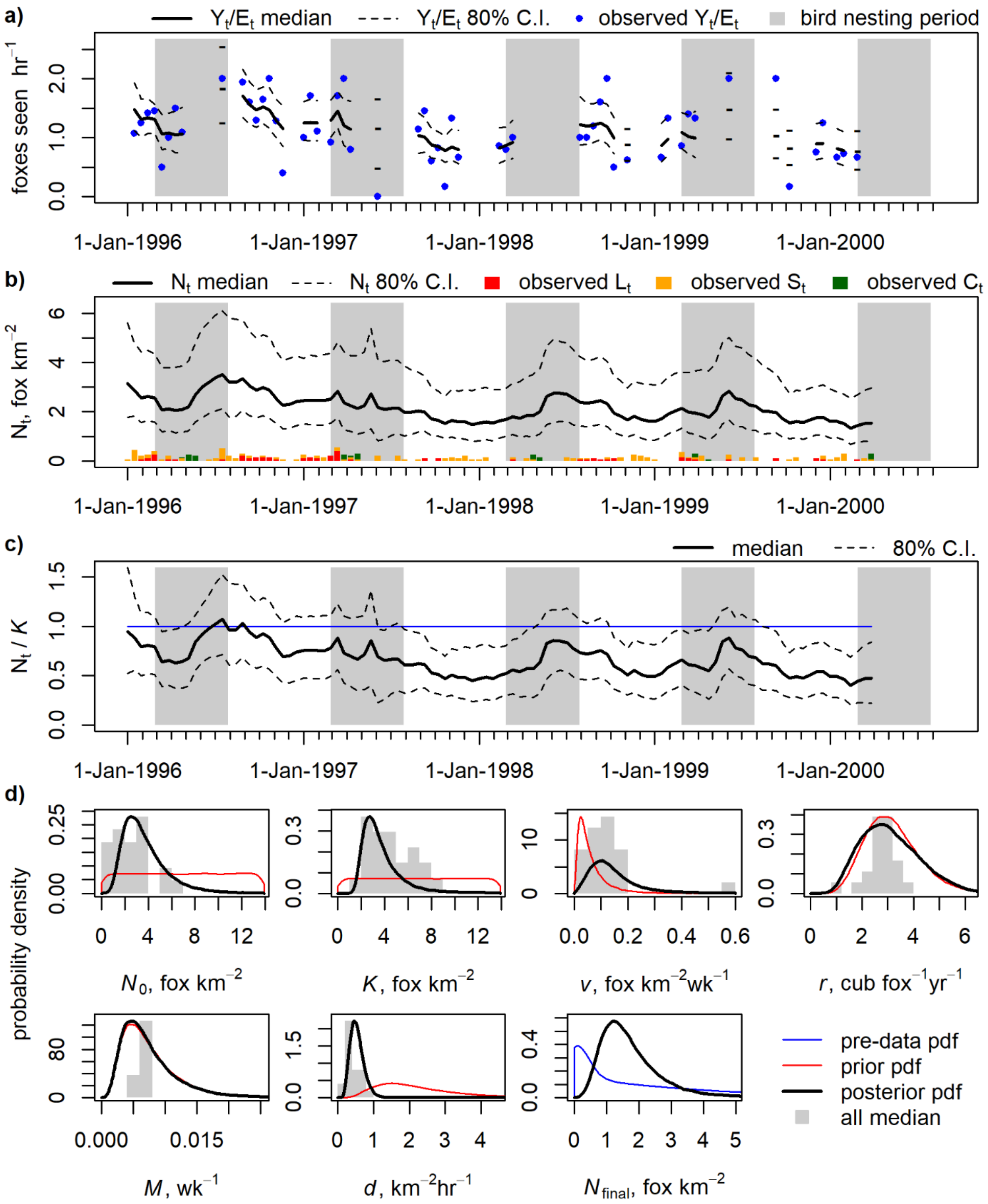

Fig A. Results for CUL showing a) posterior fit of the model to detection rate (Y_t/E_t); b) posterior estimates of bi-weekly fox density (N_t) in relation to the cull removed by different methods; c) estimated N_t as a proportion of carrying capacity (blue line denotes median population at posterior median K); d) priors (or post-model-pre-data distribution) and marginal posteriors of N_0 (initial density), K (carrying capacity), v (immigration rate), r (*per capita* birth rate), M (instantaneous non-culling mortality rate), d (rate of successful search), and fox density in the final time-step. Histograms in panel d) show posterior medians from all estates. In panels a-c) the bird nesting period (March-July) is shown as a reference.

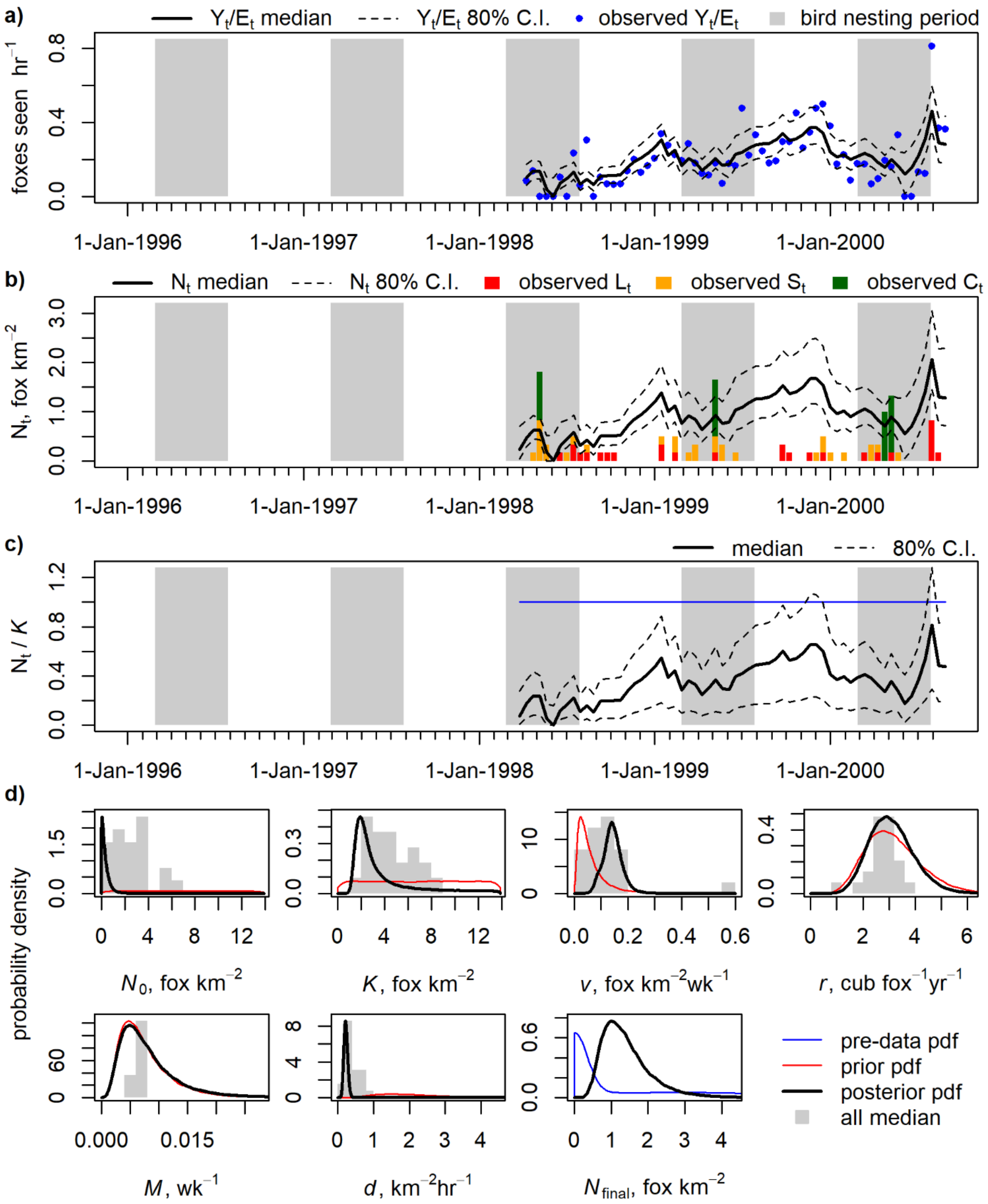

Fig B. Results for HIR showing a) posterior fit of the model to detection rate (Y_t/E_t); b) posterior estimates of bi-weekly fox density (N_t) in relation to the cull removed by different methods; c) estimated N_t as a proportion of carrying capacity (blue line denotes median population at posterior median K); d) priors (or post-model-pre-data distribution) and marginal posteriors of N_0 (initial density), K (carrying capacity), v (immigration rate), r (*per capita* birth rate), M (instantaneous non-culling mortality rate), d (rate of successful search), and fox density in the final time-step. Histograms in panel d) show posterior medians from all estates. In panels a-c) the bird nesting period (March-July) is shown as a reference.

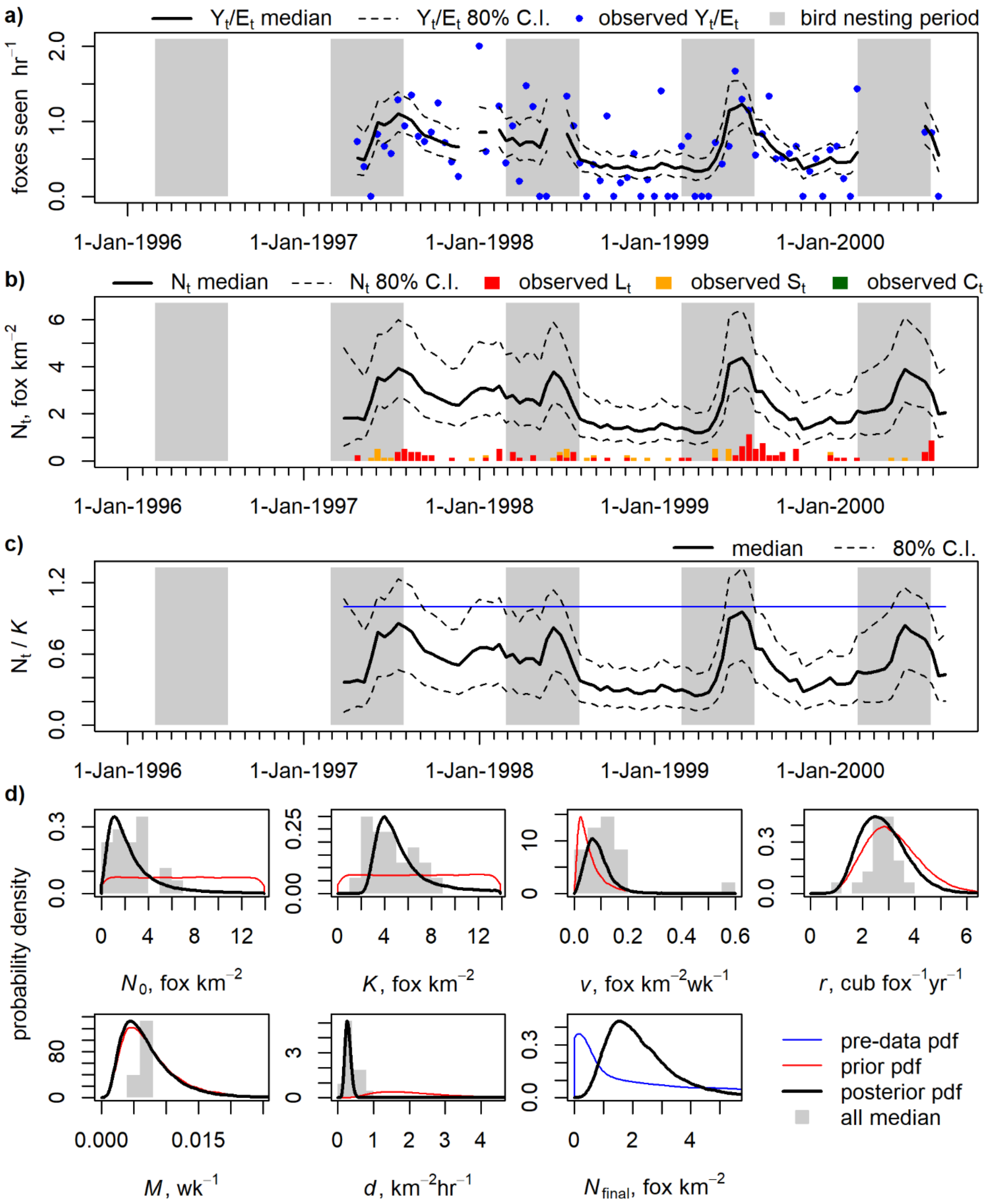

Fig C. Results for NOG showing a) posterior fit of the model to detection rate (Y_t/E_t); b) posterior estimates of bi-weekly fox density (N_t) in relation to the cull removed by different methods; c) estimated N_t as a proportion of carrying capacity (blue line denotes median population at posterior median K); d) priors (or post-model-pre-data distribution) and marginal posteriors of N_0 (initial density), K (carrying capacity), v (immigration rate), r (*per capita* birth rate), M (instantaneous non-culling mortality rate), d (rate of successful search), and fox density in the final time-step. Histograms in panel d) show posterior medians from all estates. In panels a-c) the bird nesting period (March-July) is shown as a reference.

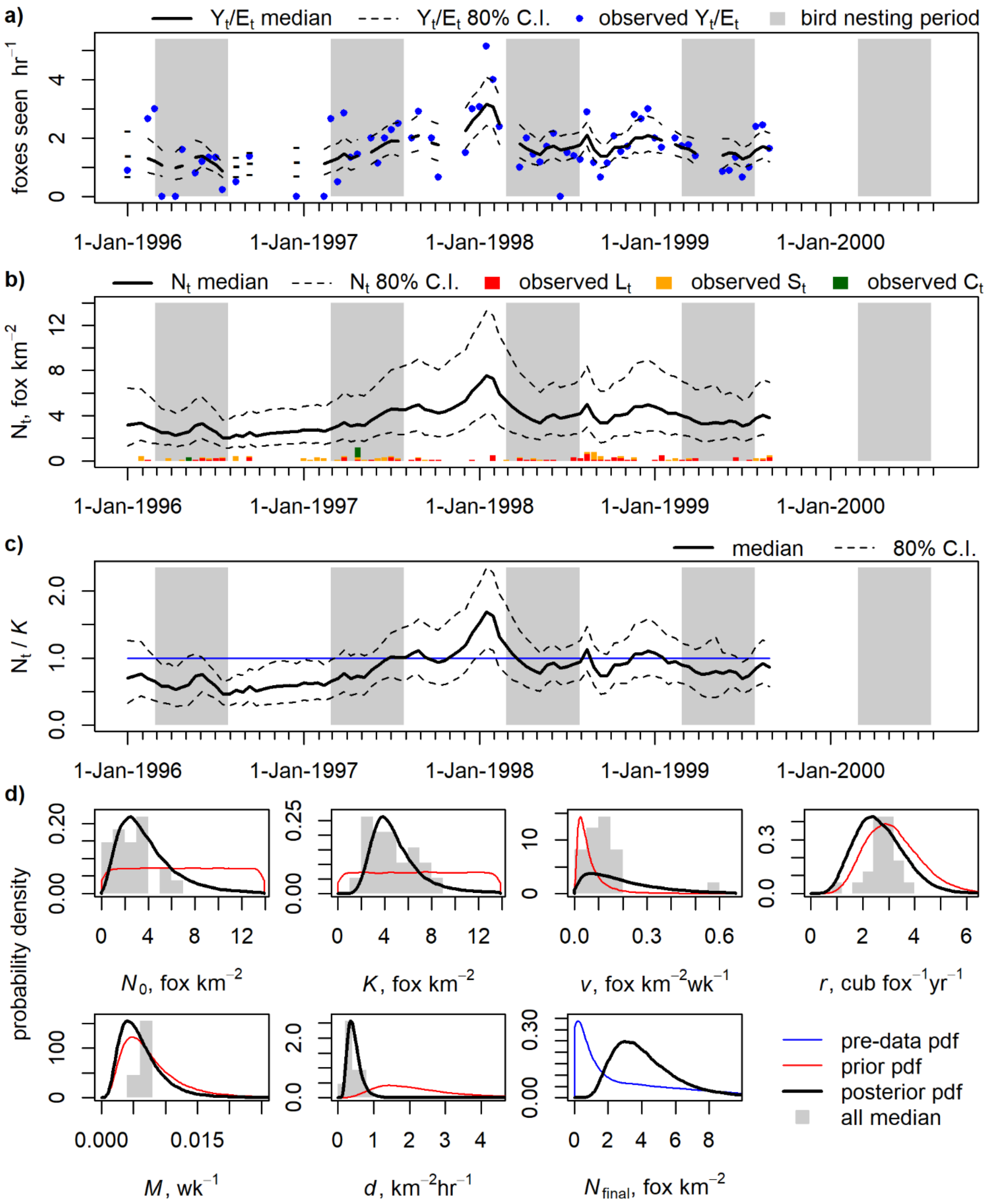

Fig D. Results for NYP showing a) posterior fit of the model to detection rate (Y_t/E_t); b) posterior estimates of bi-weekly fox density (N_t) in relation to the cull removed by different methods; c) estimated N_t as a proportion of carrying capacity (blue line denotes median population at posterior median K); d) priors (or post-model-pre-data distribution) and marginal posteriors of N_0 (initial density), K (carrying capacity), v (immigration rate), r (*per capita* birth rate), M (instantaneous non-culling mortality rate), d (rate of successful search), and fox density in the final time-step. Histograms in panel d) show posterior medians from all estates. In panels a-c) the bird nesting period (March-July) is shown as a reference.

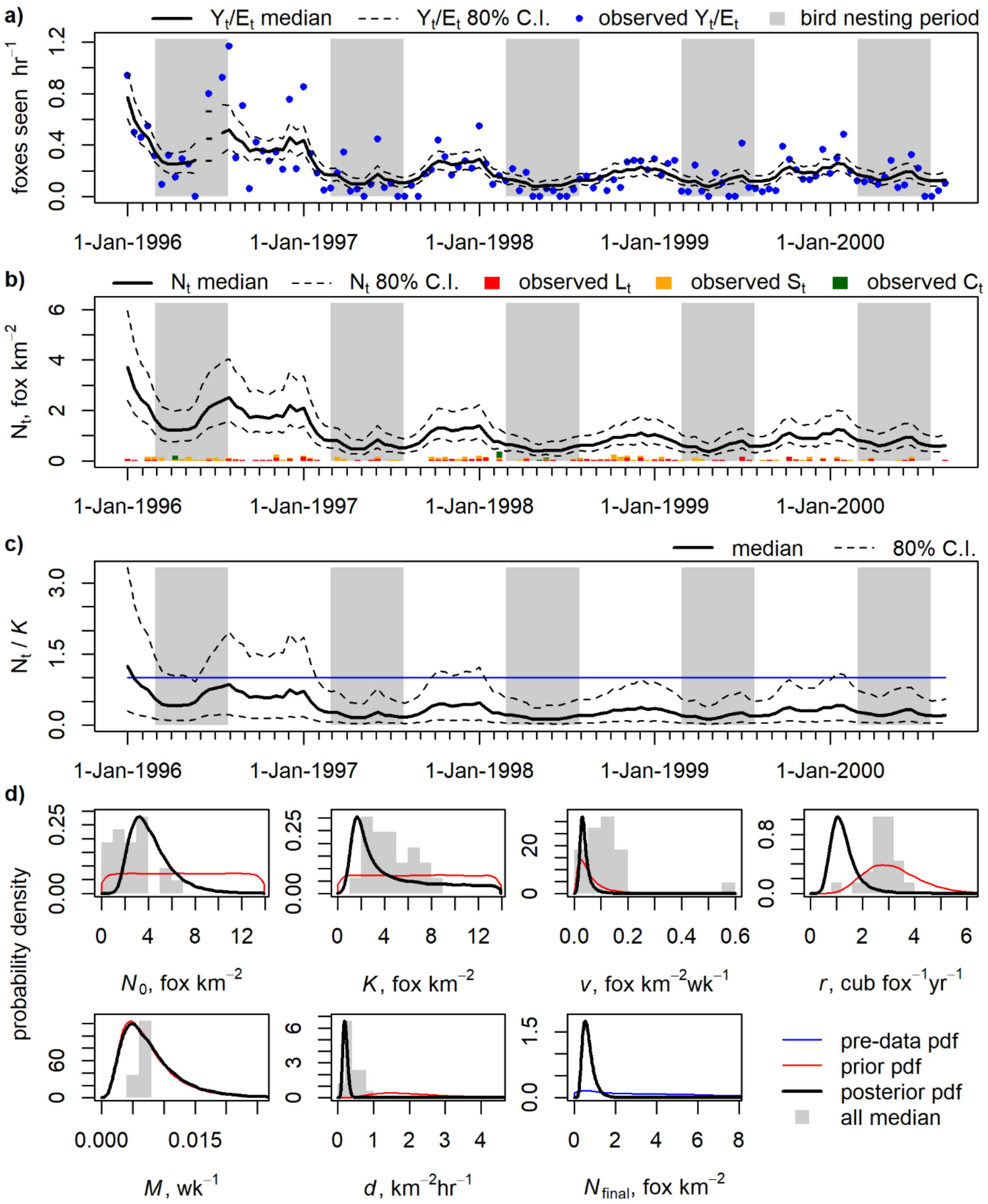

Fig E. Results for VAR showing a) posterior fit of the model to detection rate (Y_t/E_t); b) posterior estimates of bi-weekly fox density (N_t) in relation to the cull removed by different methods; c) estimated N_t as a proportion of carrying capacity (blue line denotes median population at posterior median K); d) priors (or post-model-pre-data distribution) and marginal posteriors of N_0 (initial density), K (carrying capacity), v (immigration rate), r (*per capita* birth rate), M (instantaneous non-culling mortality rate), d (rate of successful search), and fox density in the final time-step. Histograms in panel d) show posterior medians from all estates. In panels a-c) the bird nesting period (March-July) is shown as a reference.

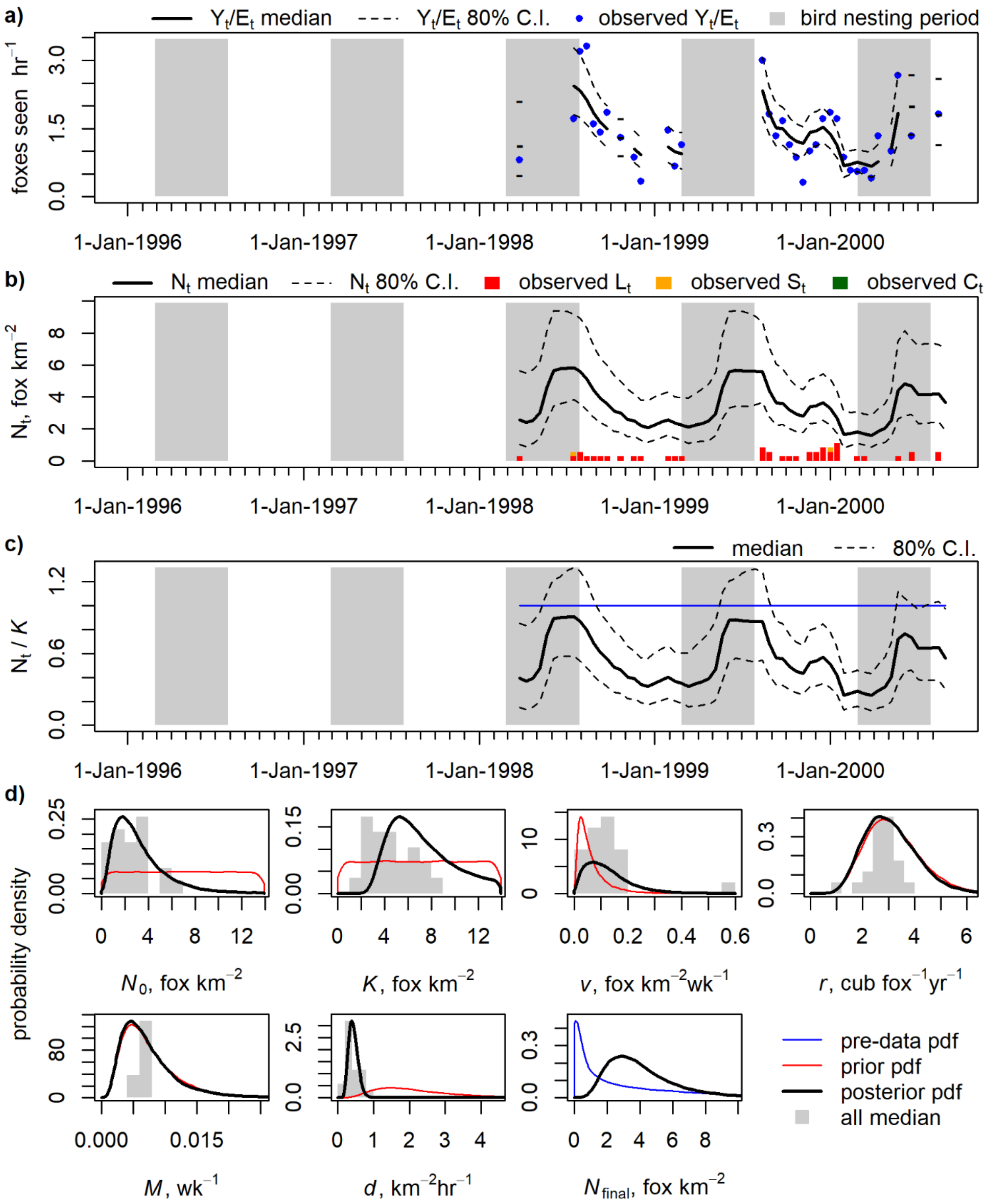

Fig F. Results for BMM showing a) posterior fit of the model to detection rate (Y_t/E_t); b) posterior estimates of bi-weekly fox density (N_t) in relation to the cull removed by different methods; c) estimated N_t as a proportion of carrying capacity (blue line denotes median population at posterior median K); d) priors (or post-model-pre-data distribution) and marginal posteriors of N_0 (initial density), K (carrying capacity), v (immigration rate), r (*per capita* birth rate), M (instantaneous non-culling mortality rate), d (rate of successful search), and fox density in the final time-step. Histograms in panel d) show posterior medians from all estates. In panels a)-c) the bird nesting period (March-July) is shown as a reference.

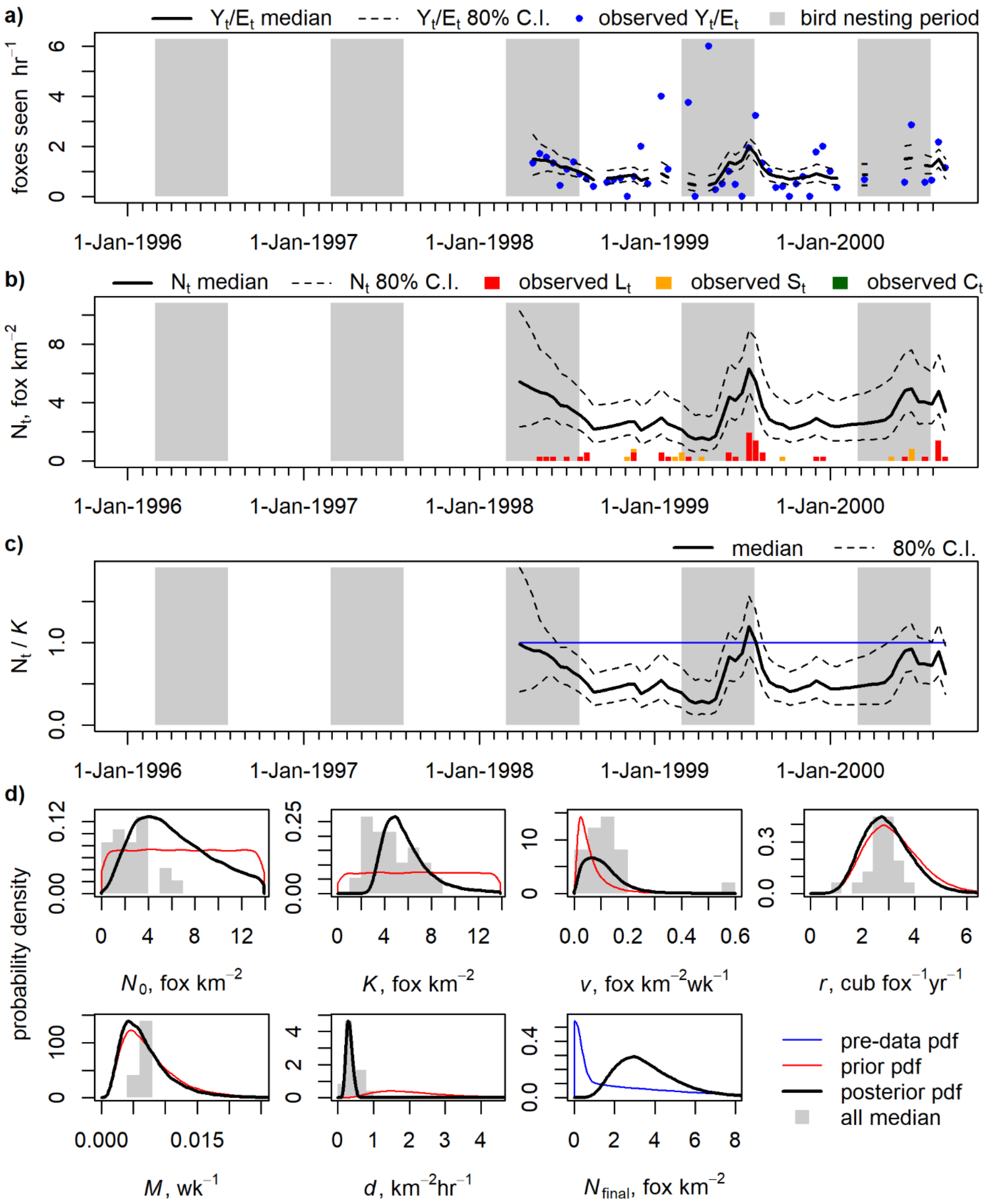

Fig G. Results for CHU showing a) posterior fit of the model to detection rate (Y_t/E_t); b) posterior estimates of bi-weekly fox density (N_t) in relation to the cull removed by different methods; c) estimated N_t as a proportion of carrying capacity (blue line denotes median population at posterior median K); d) priors (or post-model-pre-data distribution) and marginal posteriors of N_0 (initial density), K (carrying capacity), v (immigration rate), r (*per capita* birth rate), M (instantaneous non-culling mortality rate), d (rate of successful search), and fox density in the final time-step. Histograms in panel d) show posterior medians from all estates. In panels a-c) the bird nesting period (March-July) is shown as a reference.

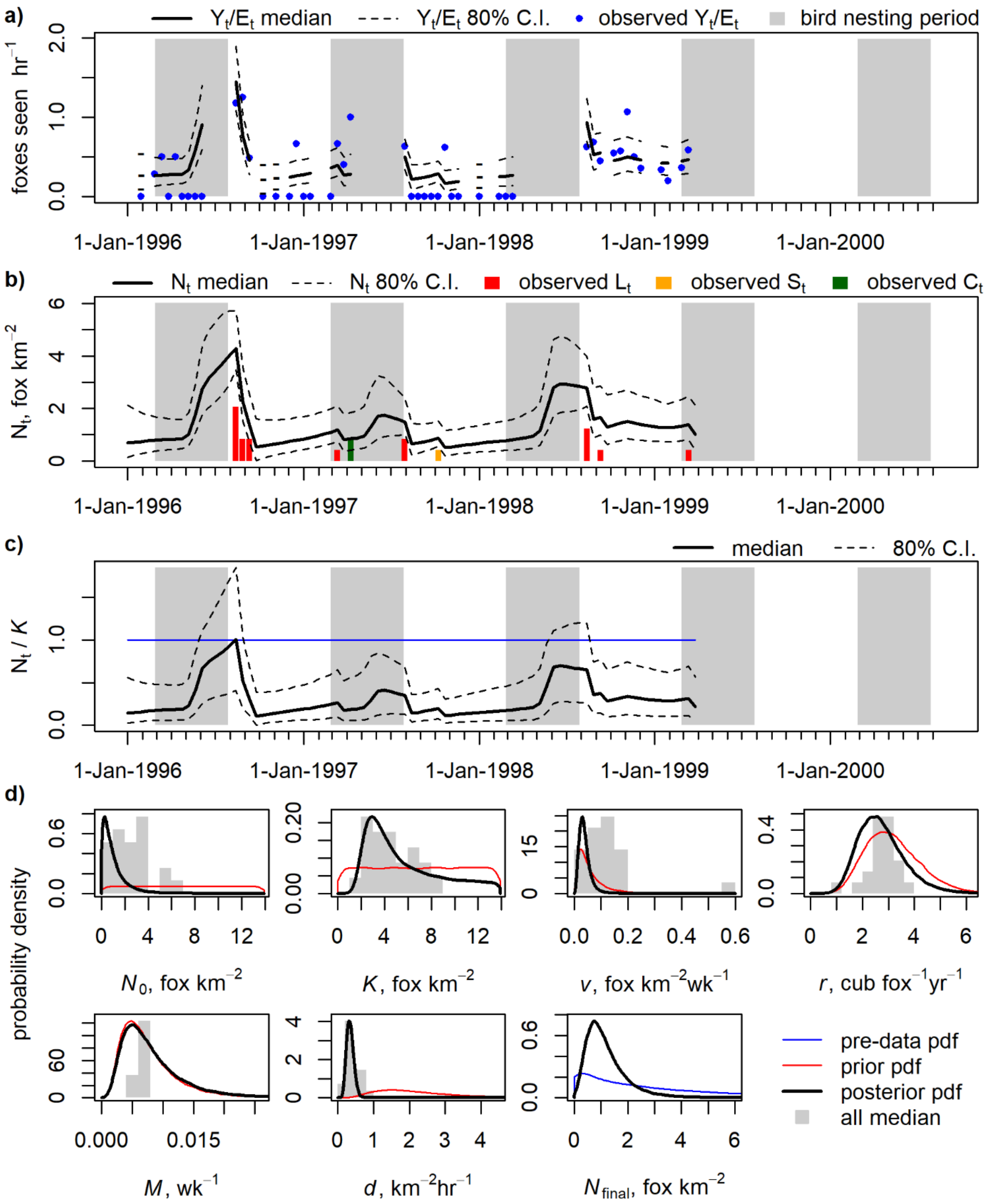

Fig H. Results for CIP showing a) posterior fit of the model to detection rate (Y_t/E_t); b) posterior estimates of bi-weekly fox density (N_t) in relation to the cull removed by different methods; c) estimated N_t as a proportion of carrying capacity (blue line denotes median population at posterior median K); d) priors (or post-model-pre-data distribution) and marginal posteriors of N_0 (initial density), K (carrying capacity), v (immigration rate), r (*per capita* birth rate), M (instantaneous non-culling mortality rate), d (rate of successful search), and fox density in the final time-step. Histograms in panel d) show posterior medians from all estates. In panels a-c) the bird nesting period (March-July) is shown as a reference.

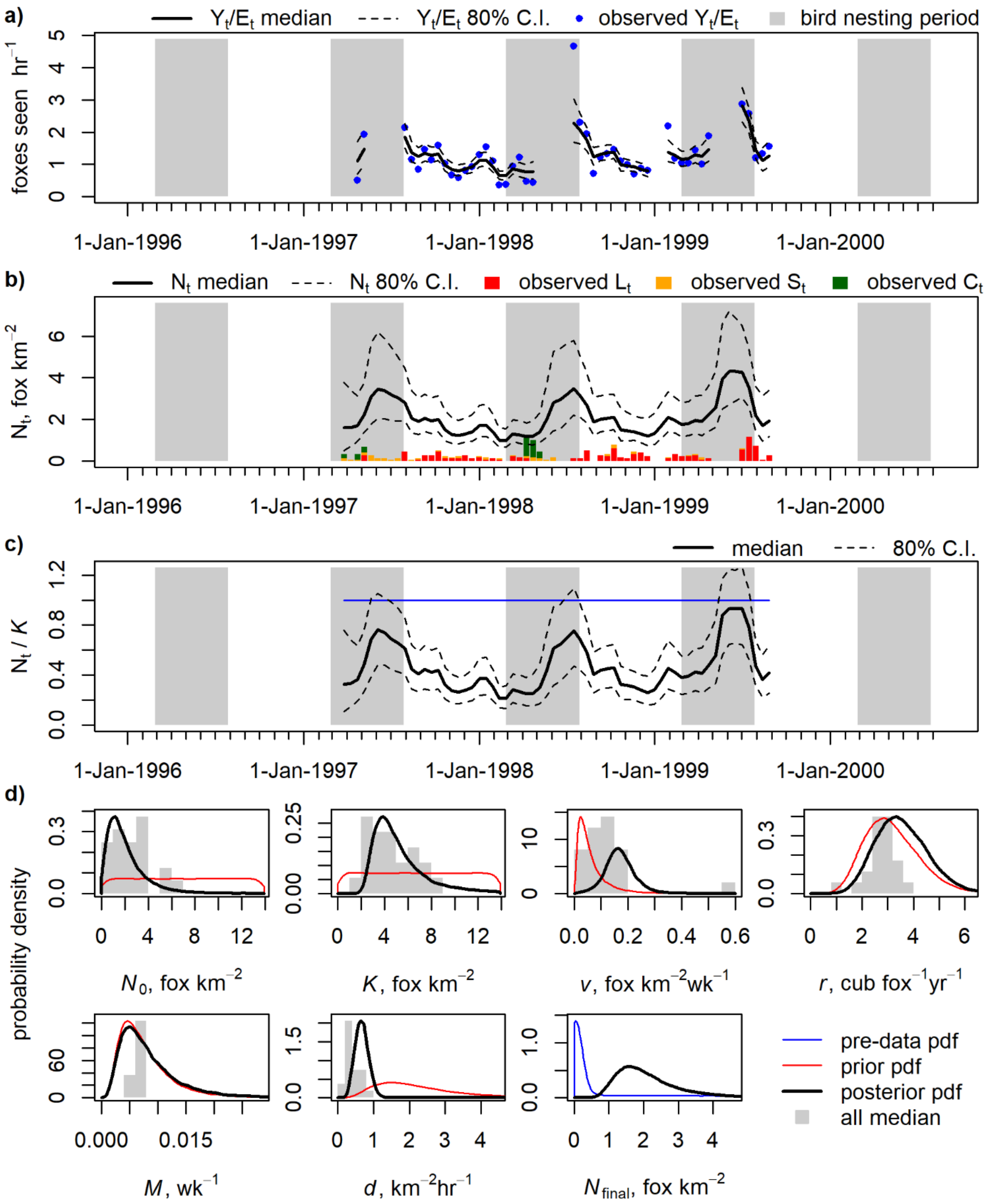

Fig I. Results for DWS showing a) posterior fit of the model to detection rate (Y_t/E_t); b) posterior estimates of bi-weekly fox density (N_t) in relation to the cull removed by different methods; c) estimated N_t as a proportion of carrying capacity (blue line denotes median population at posterior median *K*); d) priors (or post-model-pre-data distribution) and marginal posteriors of N_0 (initial density), *K* (carrying capacity), *v* (immigration rate), *r* (*per capita* birth rate), *M* (instantaneous non-culling mortality rate), *d* (rate of successful search), and fox density in the final time-step. Histograms in panel d) show posterior medians from all estates. In panels a-c) the bird nesting period (March-July) is shown as a reference.

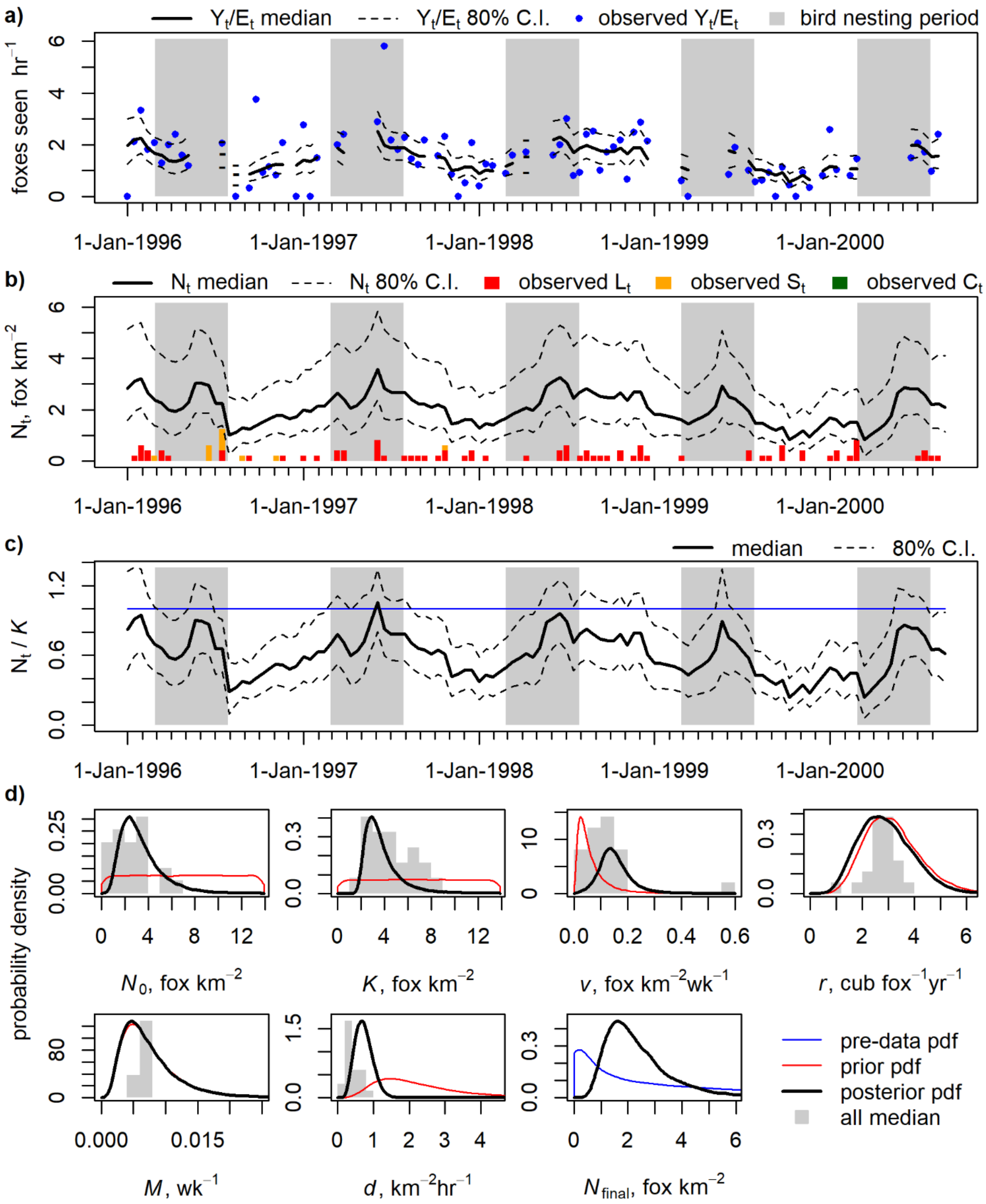

Fig J. Results for EWE showing a) posterior fit of the model to detection rate (Y_t/E_t); b) posterior estimates of bi-weekly fox density (N_t) in relation to the cull removed by different methods; c) estimated N_t as a proportion of carrying capacity (blue line denotes median population at posterior median K); d) priors (or post-model-pre-data distribution) and marginal posteriors of N_0 (initial density), K (carrying capacity), v (immigration rate), r (*per capita* birth rate), M (instantaneous non-culling mortality rate), d (rate of successful search), and fox density in the final time-step. Histograms in panel d) show posterior medians from all estates. In panels a-c) the bird nesting period (March-July) is shown as a reference.

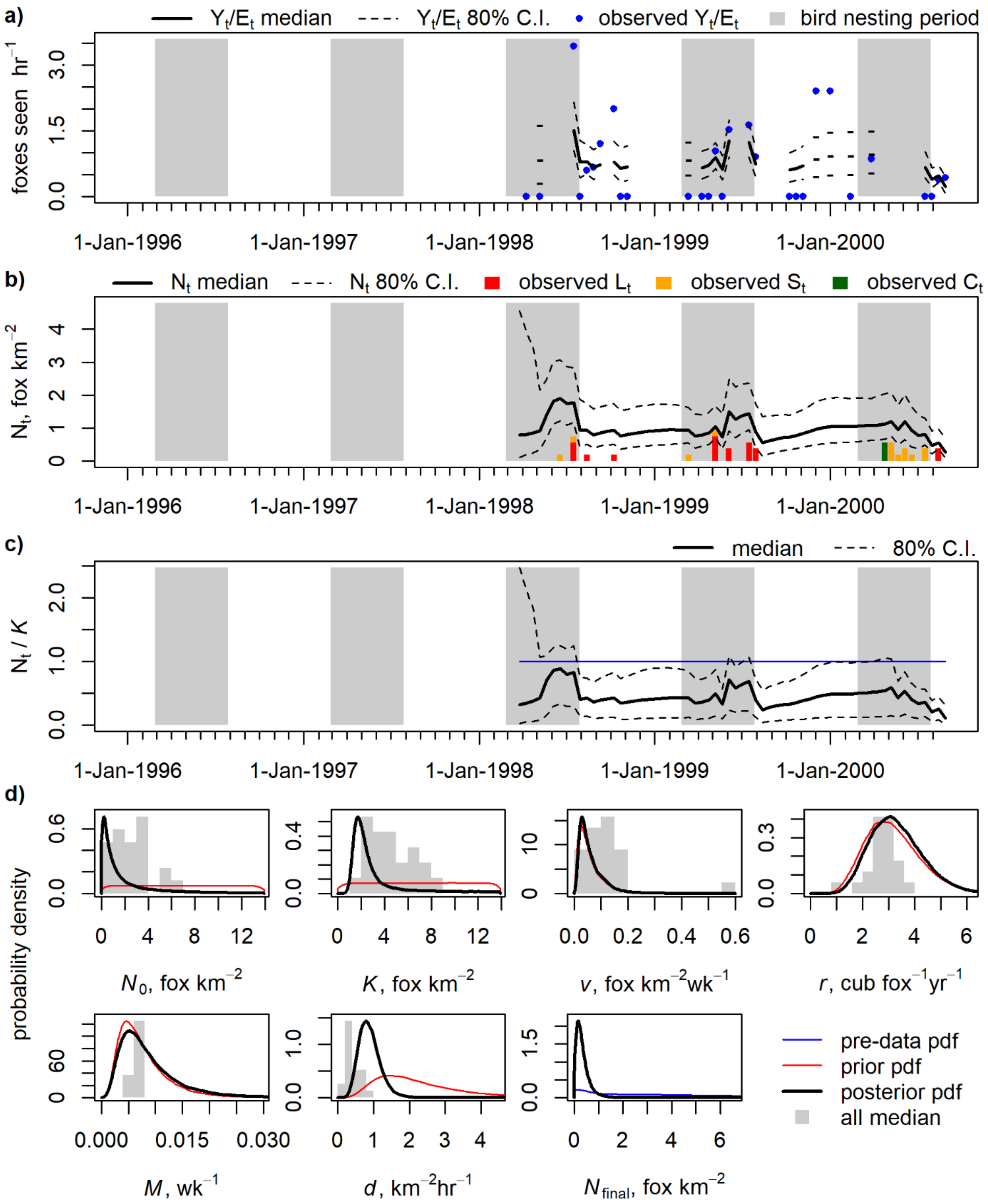

Fig K. Results for FAH showing a) posterior fit of the model to detection rate (Y_t/E_t); b) posterior estimates of bi-weekly fox density (N_t) in relation to the cull removed by different methods; c) estimated N_t as a proportion of carrying capacity (blue line denotes median population at posterior median K); d) priors (or post-model-pre-data distribution) and marginal posteriors of N_0 (initial density), K (carrying capacity), v (immigration rate), r (*per capita* birth rate), M (instantaneous non-culling mortality rate), d (rate of successful search), and fox density in the final time-step. Histograms in panel d) show posterior medians from all estates. In panels a-c) the bird nesting period (March-July) is shown as a reference.

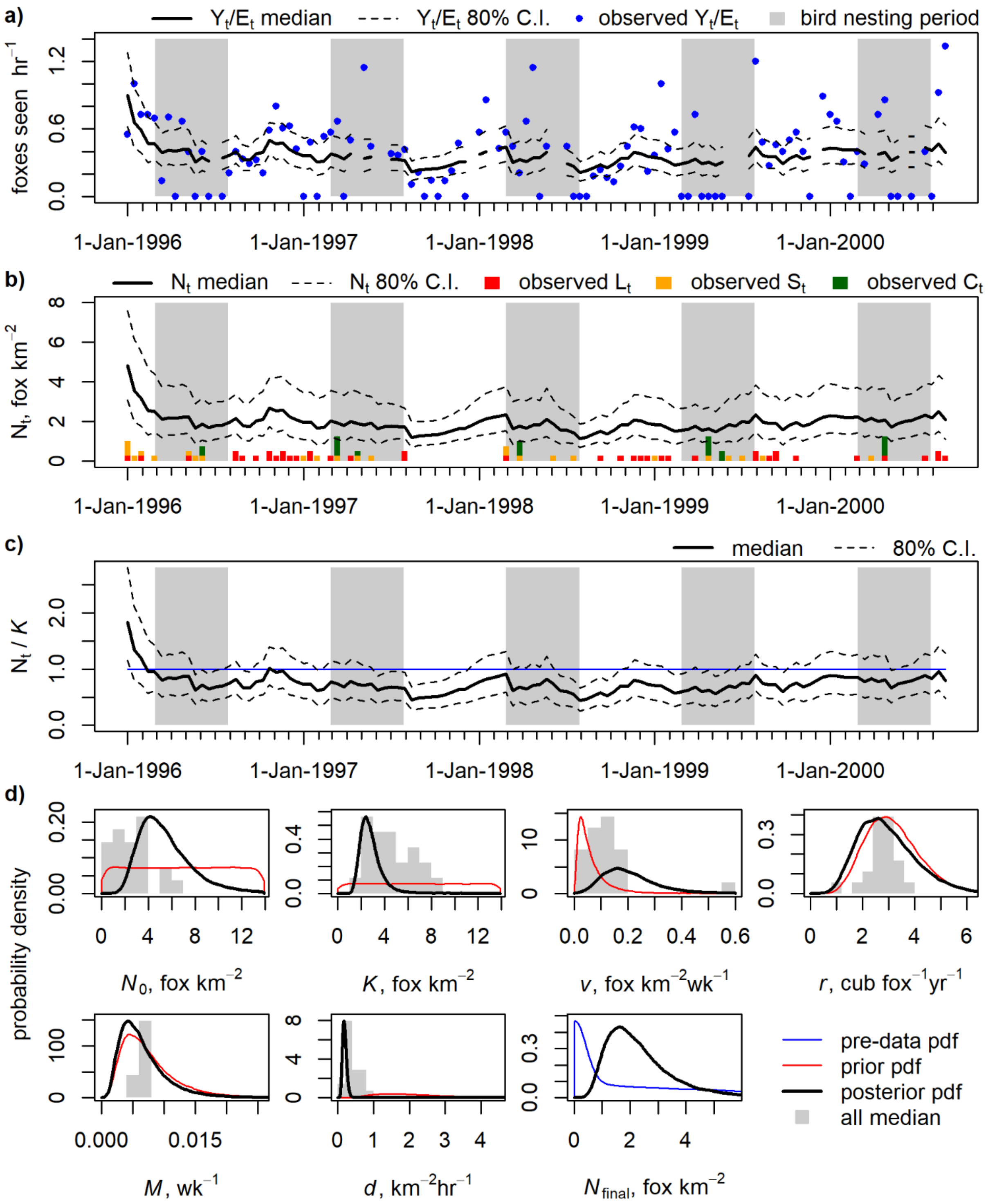

Fig L. Results for FHC showing a) posterior fit of the model to detection rate (Y_t/E_t); b) posterior estimates of bi-weekly fox density (N_t) in relation to the cull removed by different methods; c) estimated N_t as a proportion of carrying capacity (blue line denotes median population at posterior median K); d) priors (or post-model-pre-data distribution) and marginal posteriors of N_0 (initial density), K (carrying capacity), v (immigration rate), r (*per capita* birth rate), M (instantaneous non-culling mortality rate), d (rate of successful search), and fox density in the final time-step. Histograms in panel d) show posterior medians from all estates. In panels a-c) the bird nesting period (March-July) is shown as a reference.

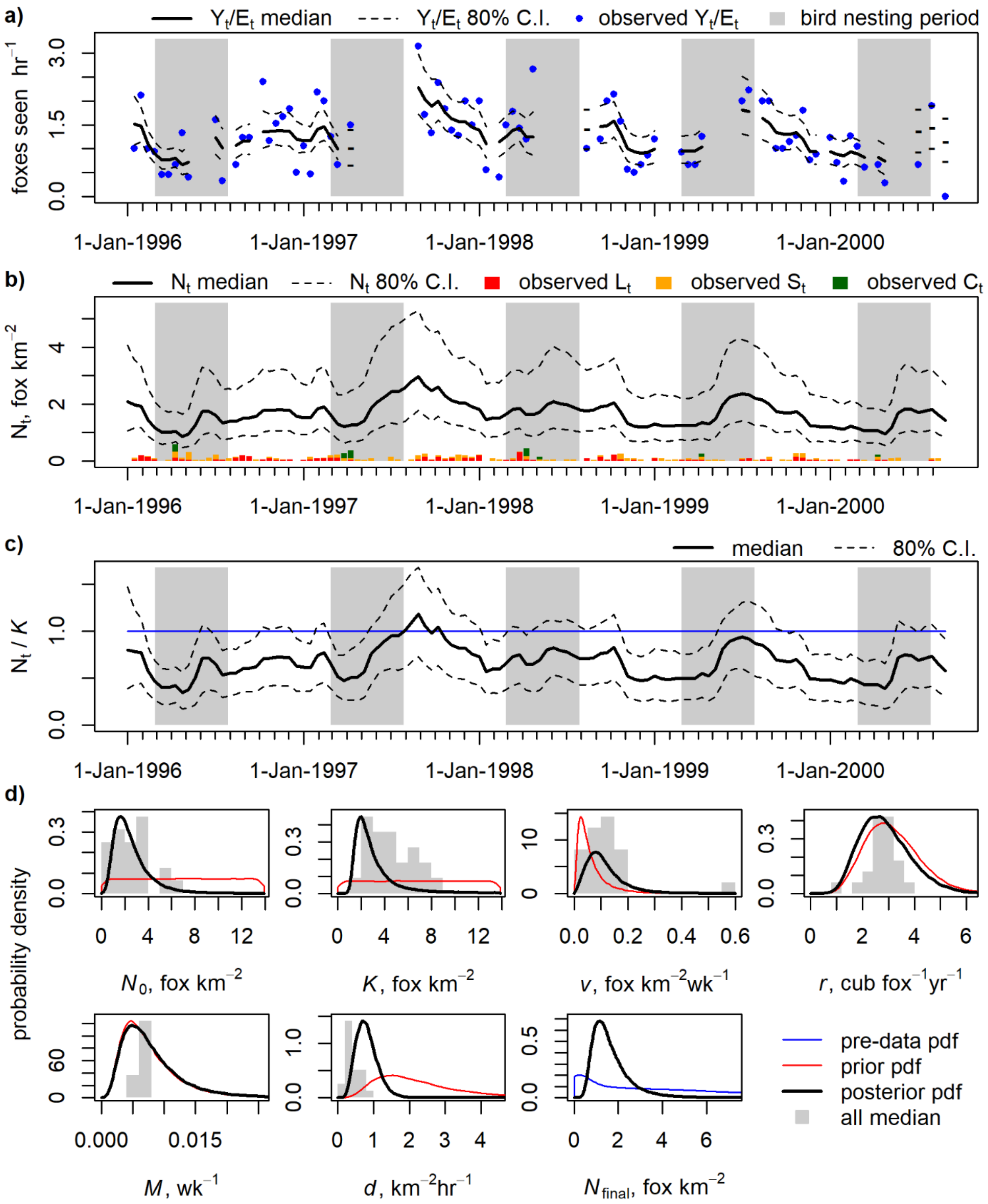

Fig M. Results for GDE showing a) posterior fit of the model to detection rate (Y_t/E_t); b) posterior estimates of bi-weekly fox density (N_t) in relation to the cull removed by different methods; c) estimated N_t as a proportion of carrying capacity (blue line denotes median population at posterior median K); d) priors (or post-model-pre-data distribution) and marginal posteriors of N_0 (initial density), K (carrying capacity), v (immigration rate), r (*per capita* birth rate), M (instantaneous non-culling mortality rate), d (rate of successful search), and fox density in the final time-step. Histograms in panel d) show posterior medians from all estates. In panels a-c) the bird nesting period (March-July) is shown as a reference.

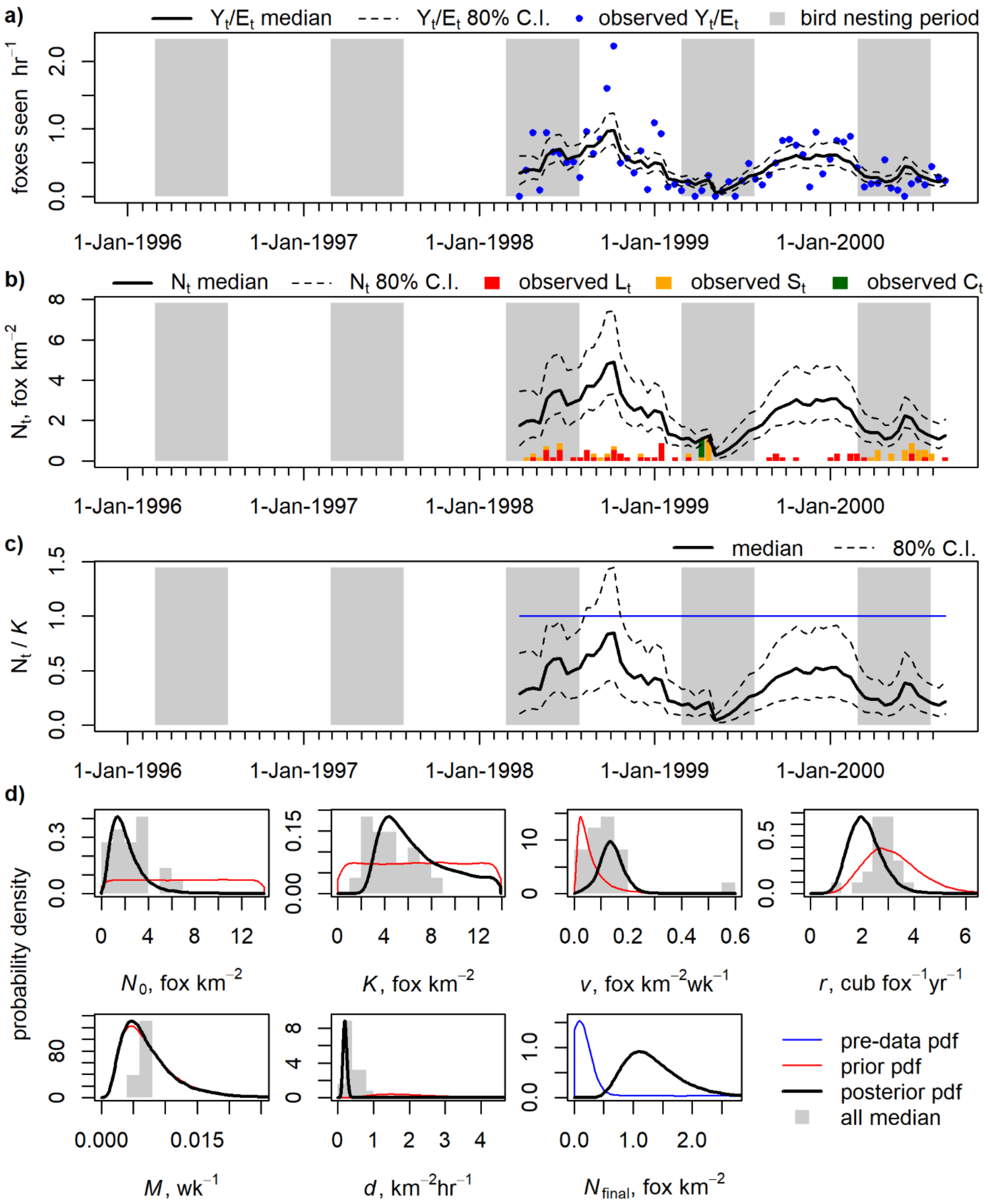

Fig N. Results for GHT showing a) posterior fit of the model to detection rate (Y_t/E_t); b) posterior estimates of bi-weekly fox density (N_t) in relation to the cull removed by different methods; c) estimated N_t as a proportion of carrying capacity (blue line denotes median population at posterior median K); d) priors (or post-model-pre-data distribution) and marginal posteriors of N_0 (initial density), K (carrying capacity), v (immigration rate), r (*per capita* birth rate), M (instantaneous non-culling mortality rate), d (rate of successful search), and fox density in the final time-step. Histograms in panel d) show posterior medians from all estates. In panels a-c) the bird nesting period (March-July) is shown as a reference.

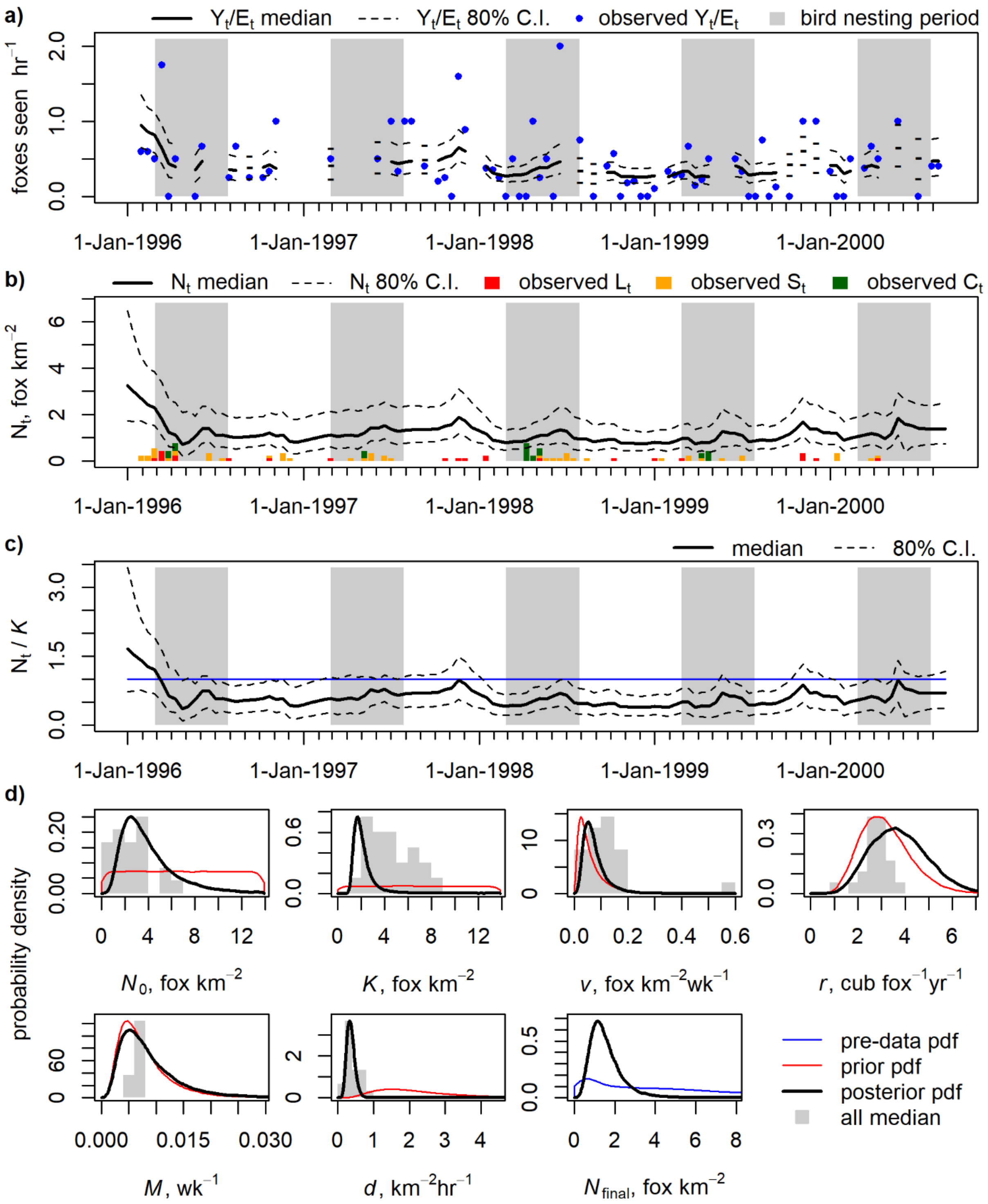

Fig O. Results for HUS showing a) posterior fit of the model to detection rate (Y_t/E_t); b) posterior estimates of bi-weekly fox density (N_t) in relation to the cull removed by different methods; c) estimated N_t as a proportion of carrying capacity (blue line denotes median population at posterior median K); d) priors (or post-model-pre-data distribution) and marginal posteriors of N_0 (initial density), K (carrying capacity), v (immigration rate), r (*per capita* birth rate), M (instantaneous non-culling mortality rate), d (rate of successful search), and fox density in the final time-step. Histograms in panel d) show posterior medians from all estates. In panels a-c) the bird nesting period (March-July) is shown as a reference.

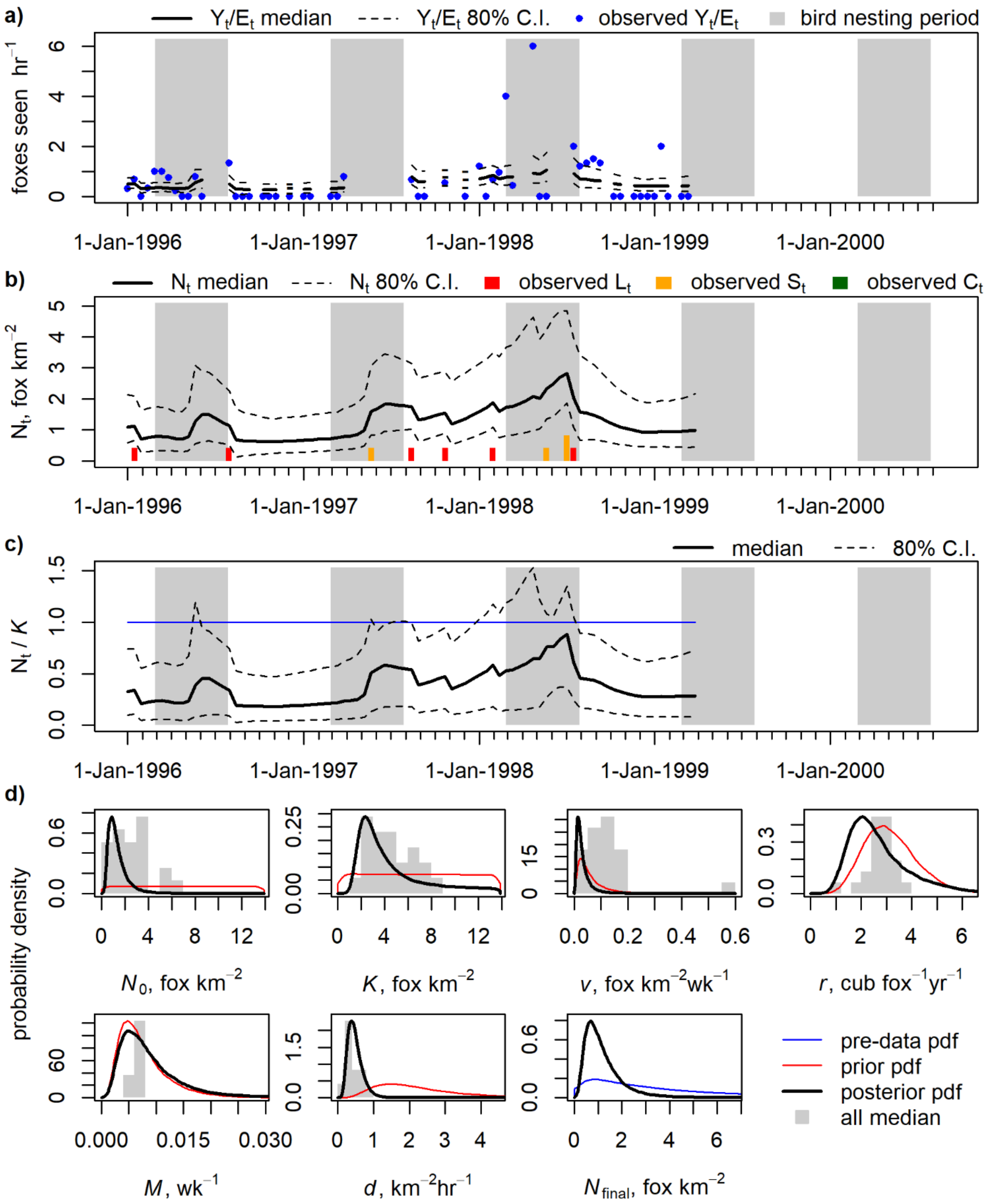

Fig P. Results for LEL showing a) posterior fit of the model to detection rate (Y_t/E_t); b) posterior estimates of bi-weekly fox density (N_t) in relation to the cull removed by different methods; c) estimated N_t as a proportion of carrying capacity (blue line denotes median population at posterior median K); d) priors (or post-model-pre-data distribution) and marginal posteriors of N_0 (initial density), K (carrying capacity), v (immigration rate), r (*per capita* birth rate), M (instantaneous non-culling mortality rate), d (rate of successful search), and fox density in the final time-step. Histograms in panel d) show posterior medians from all estates. In panels a-c) the bird nesting period (March-July) is shown as a reference.

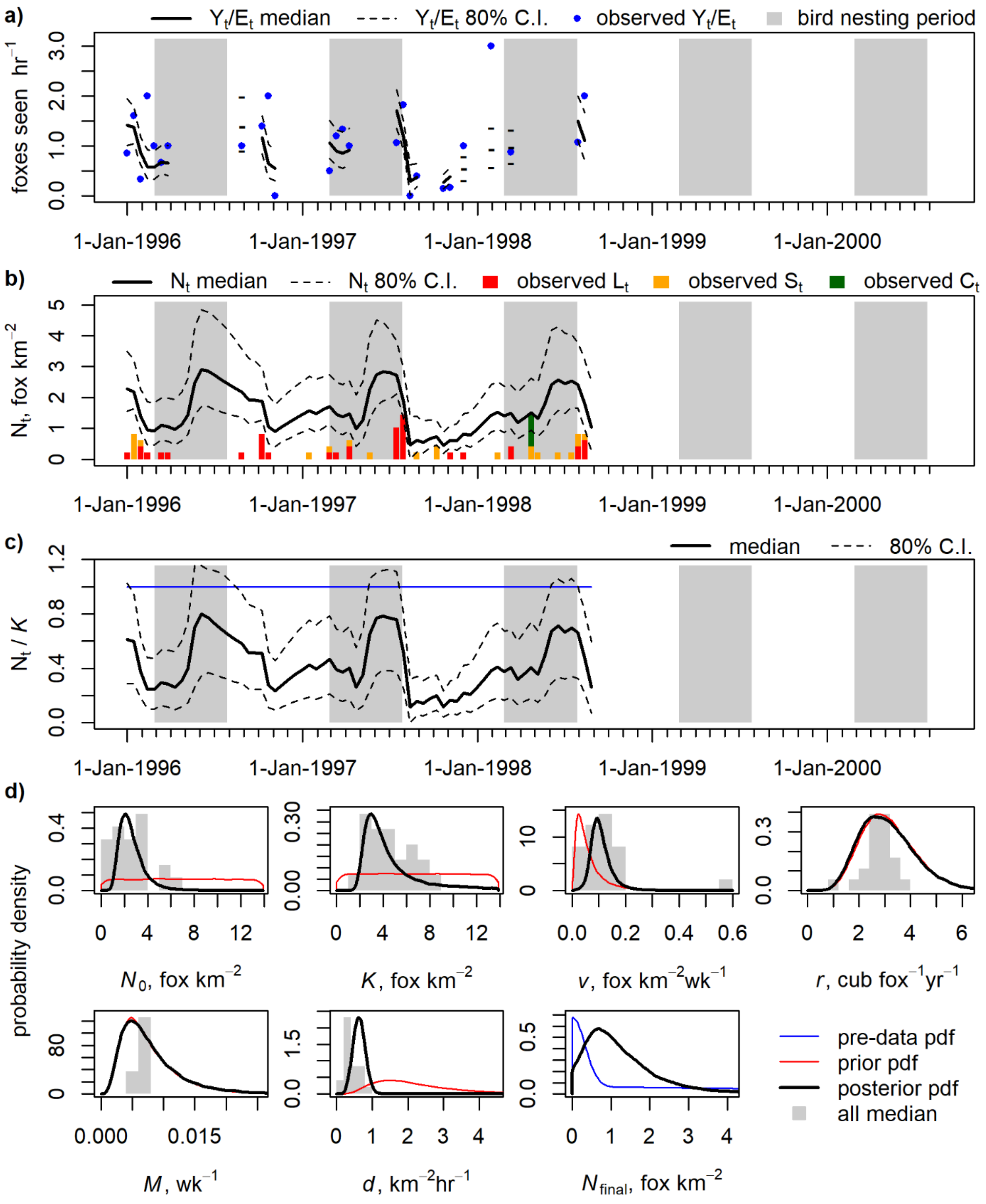

Fig Q. Results for MAH showing a) posterior fit of the model to detection rate (Y_t/E_t); b) posterior estimates of bi-weekly fox density (N_t) in relation to the cull removed by different methods; c) estimated N_t as a proportion of carrying capacity (blue line denotes median population at posterior median K); d) priors (or post-model-pre-data distribution) and marginal posteriors of N_0 (initial density), K (carrying capacity), v (immigration rate), r (*per capita* birth rate), M (instantaneous non-culling mortality rate), d (rate of successful search), and fox density in the final time-step. Histograms in panel d) show posterior medians from all estates. In panels a-c) the bird nesting period (March-July) is shown as a reference.

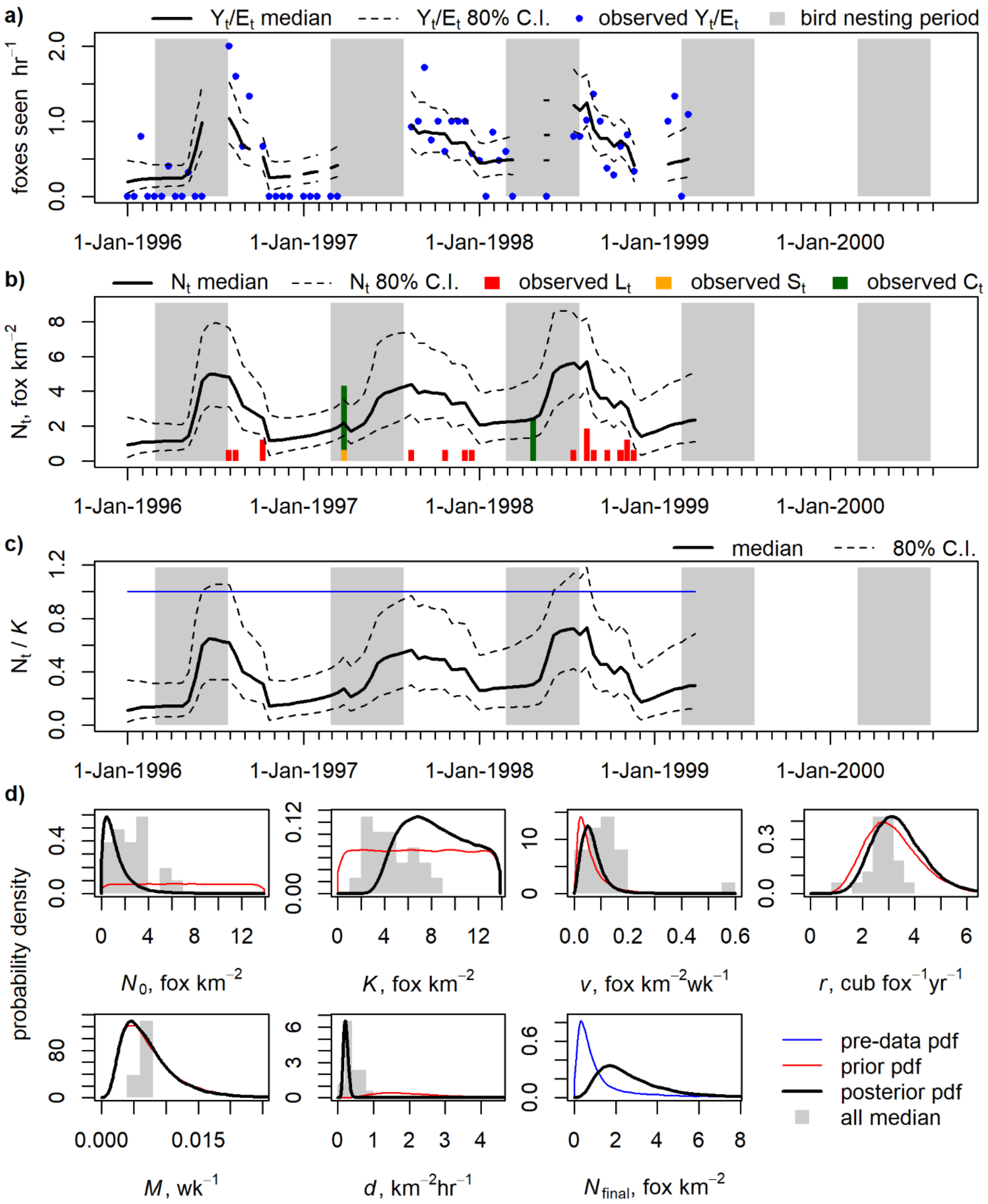

Fig R. Results for OCS showing a) posterior fit of the model to detection rate (Y_t/E_t); b) posterior estimates of bi-weekly fox density (N_t) in relation to the cull removed by different methods; c) estimated N_t as a proportion of carrying capacity (blue line denotes median population at posterior median K); d) priors (or post-model-pre-data distribution) and marginal posteriors of N_0 (initial density), K (carrying capacity), v (immigration rate), r (*per capita* birth rate), M (instantaneous non-culling mortality rate), d (rate of successful search), and fox density in the final time-step. Histograms in panel d) show posterior medians from all estates. In panels a-c) the bird nesting period (March-July) is shown as a reference.

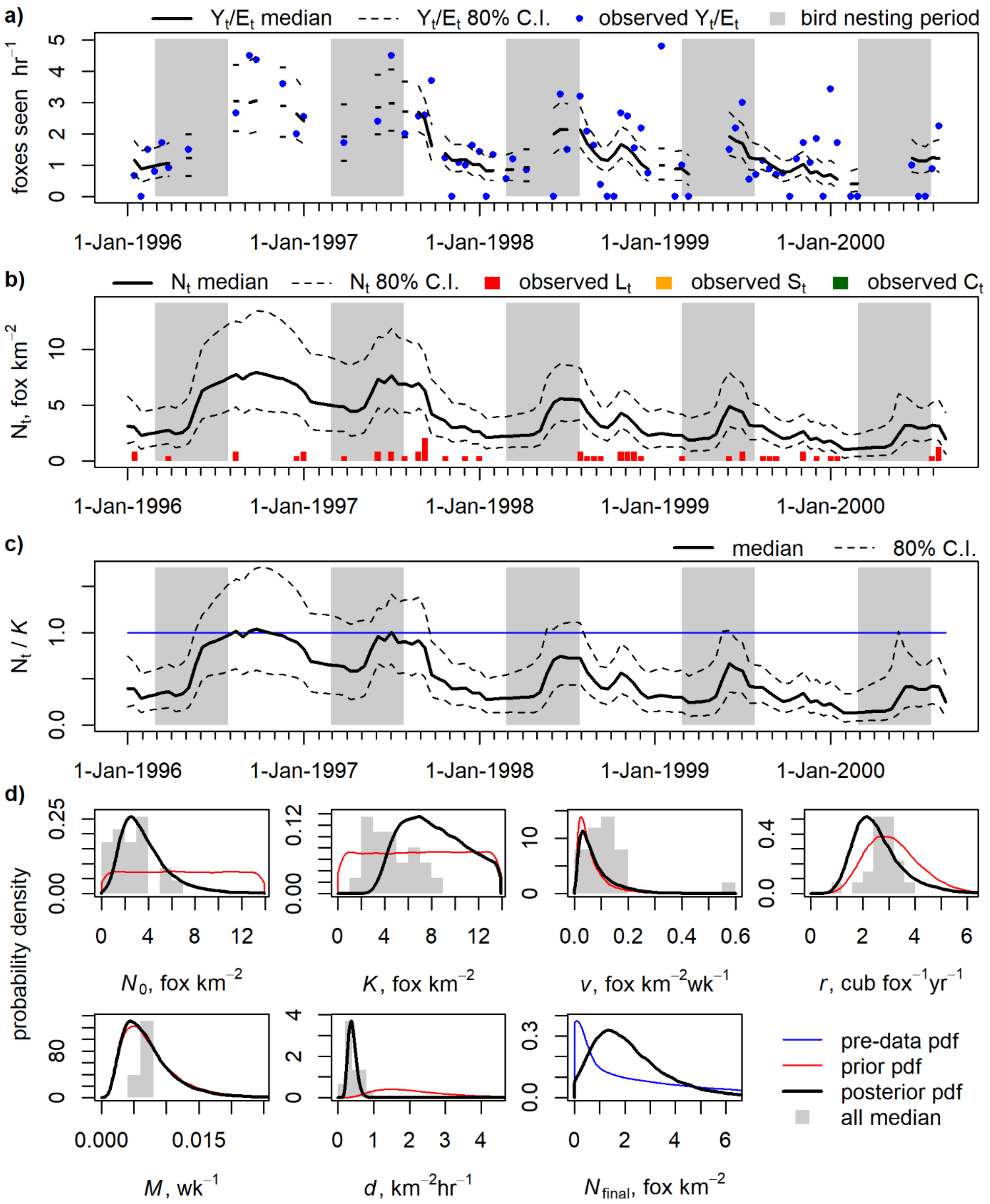

Fig S. Results for RAM showing a) posterior fit of the model to detection rate (Y_t/E_t); b) posterior estimates of bi-weekly fox density (N_t) in relation to the cull removed by different methods; c) estimated N_t as a proportion of carrying capacity (blue line denotes median population at posterior median K); d) priors (or post-model-pre-data distribution) and marginal posteriors of N_0 (initial density), K (carrying capacity), v (immigration rate), r (*per capita* birth rate), M (instantaneous non-culling mortality rate), d (rate of successful search), and fox density in the final time-step. Histograms in panel d) show posterior medians from all estates. In panels a-c) the bird nesting period (March-July) is shown as a reference.

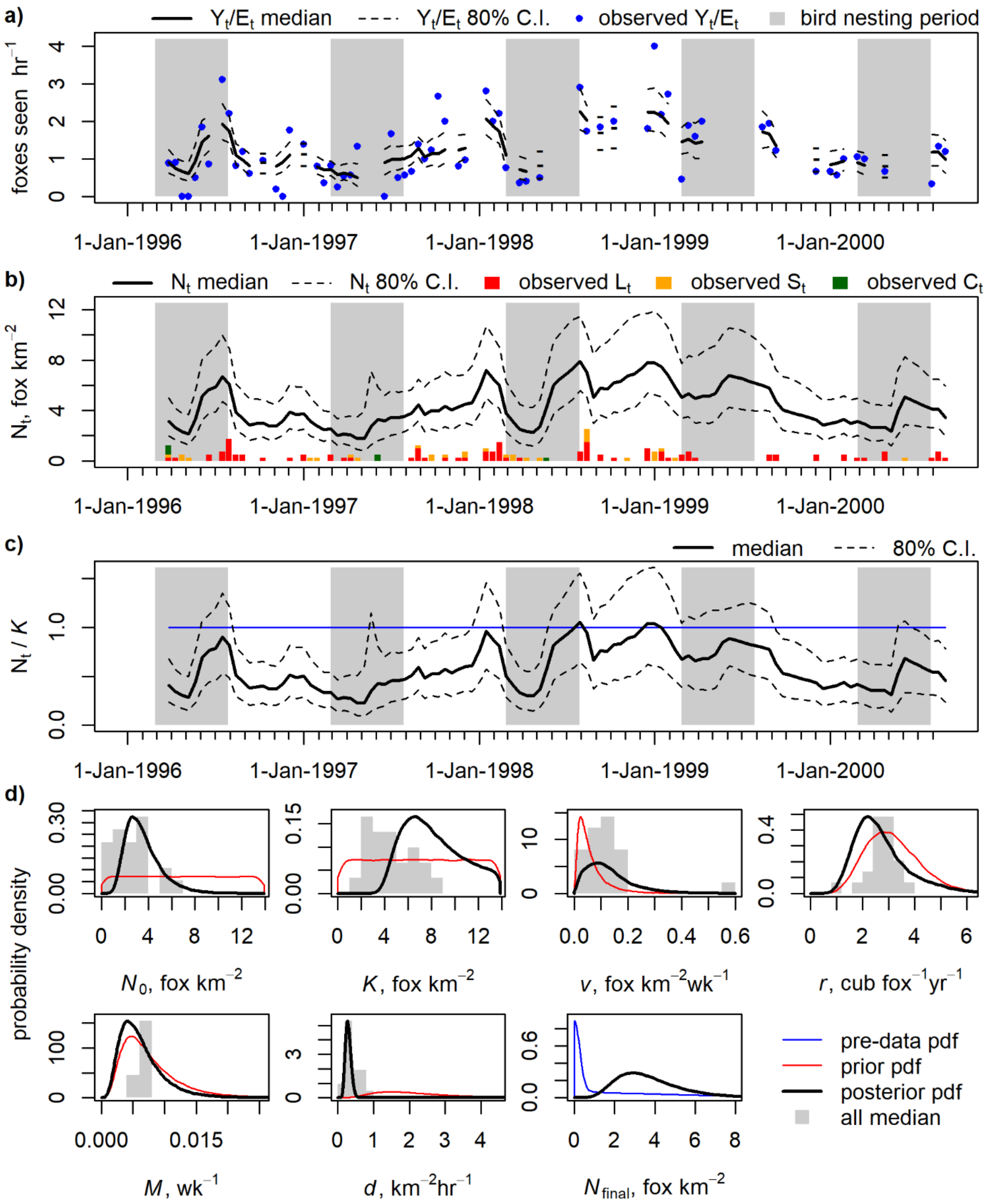

Fig T. Results for VDL showing a) posterior fit of the model to detection rate (Y_t/E_t); b) posterior estimates of bi-weekly fox density (N_t) in relation to the cull removed by different methods; c) estimated N_t as a proportion of carrying capacity (blue line denotes median population at posterior median K); d) priors (or post-model-pre-data distribution) and marginal posteriors of N_0 (initial density), K (carrying capacity), v (immigration rate), r (*per capita* birth rate), M (instantaneous non-culling mortality rate), d (rate of successful search), and fox density in the final time-step. Histograms in panel d) show posterior medians from all estates. In panels a-c) the bird nesting period (March-July) is shown as a reference.

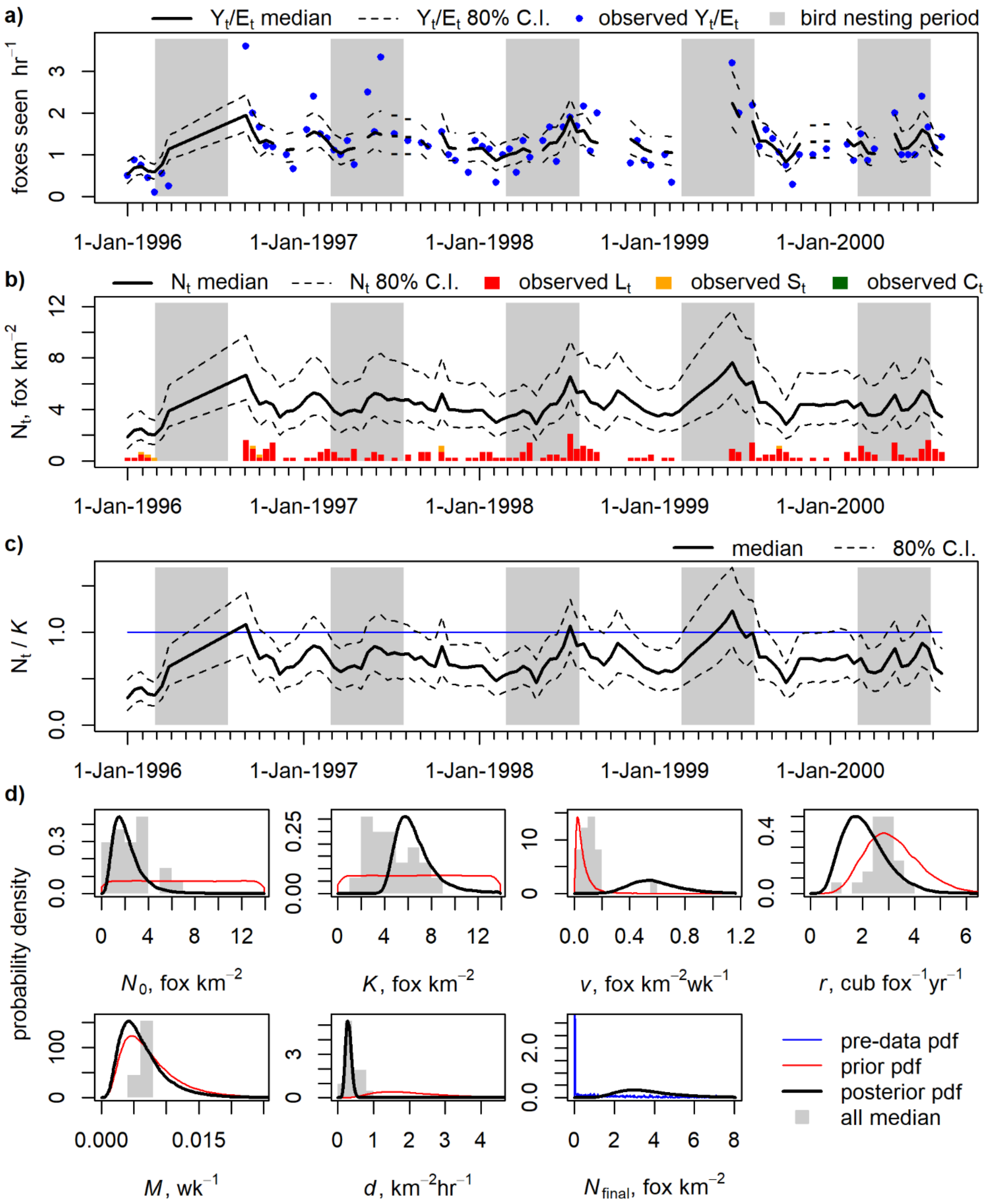

Fig U. Results for YZM showing a) posterior fit of the model to detection rate (Y_t/E_t); b) posterior estimates of bi-weekly fox density (N_t) in relation to the cull removed by different methods; c) estimated N_t as a proportion of carrying capacity (blue line denotes median population at posterior median K); d) priors (or post-model-pre-data distribution) and marginal posteriors of N_0 (initial density), K (carrying capacity), v (immigration rate), r (*per capita* birth rate), M (instantaneous non-culling mortality rate), d (rate of successful search), and fox density in the final time-step. Histograms in panel d) show posterior medians from all estates. In panels a-c) the bird nesting period (March-July) is shown as a reference.
