# Supplementary figures and images for: Population dynamics of foxes during restricted-area culling in Britain: Advancing understanding through state-space modelling of culling records
Source: PLoS One. 2019 Nov 19;14(11):e0225201. doi: 10.1371/journal.pone.0225201 (PMC6863561; doi:10.1371/journal.pone.0225201)

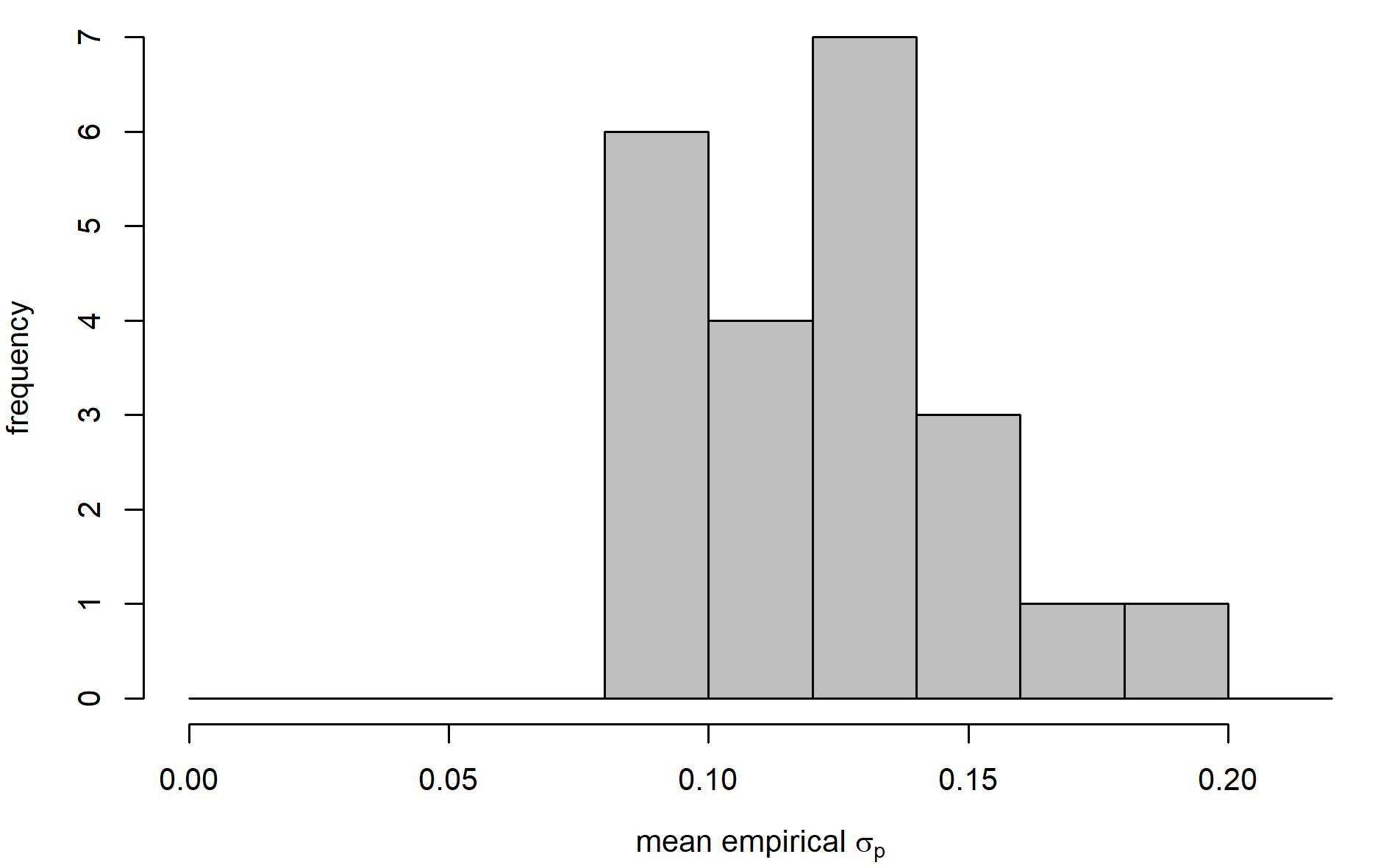

Supplement: S1 Fig — These realised values were obtained by calculating the standard deviation in process errors across the time series for each MCMC chain iteration and summarising the mean of those. The fixed value used in the model was 0.2. (TIF) [file pone.0225201.s006.tif]

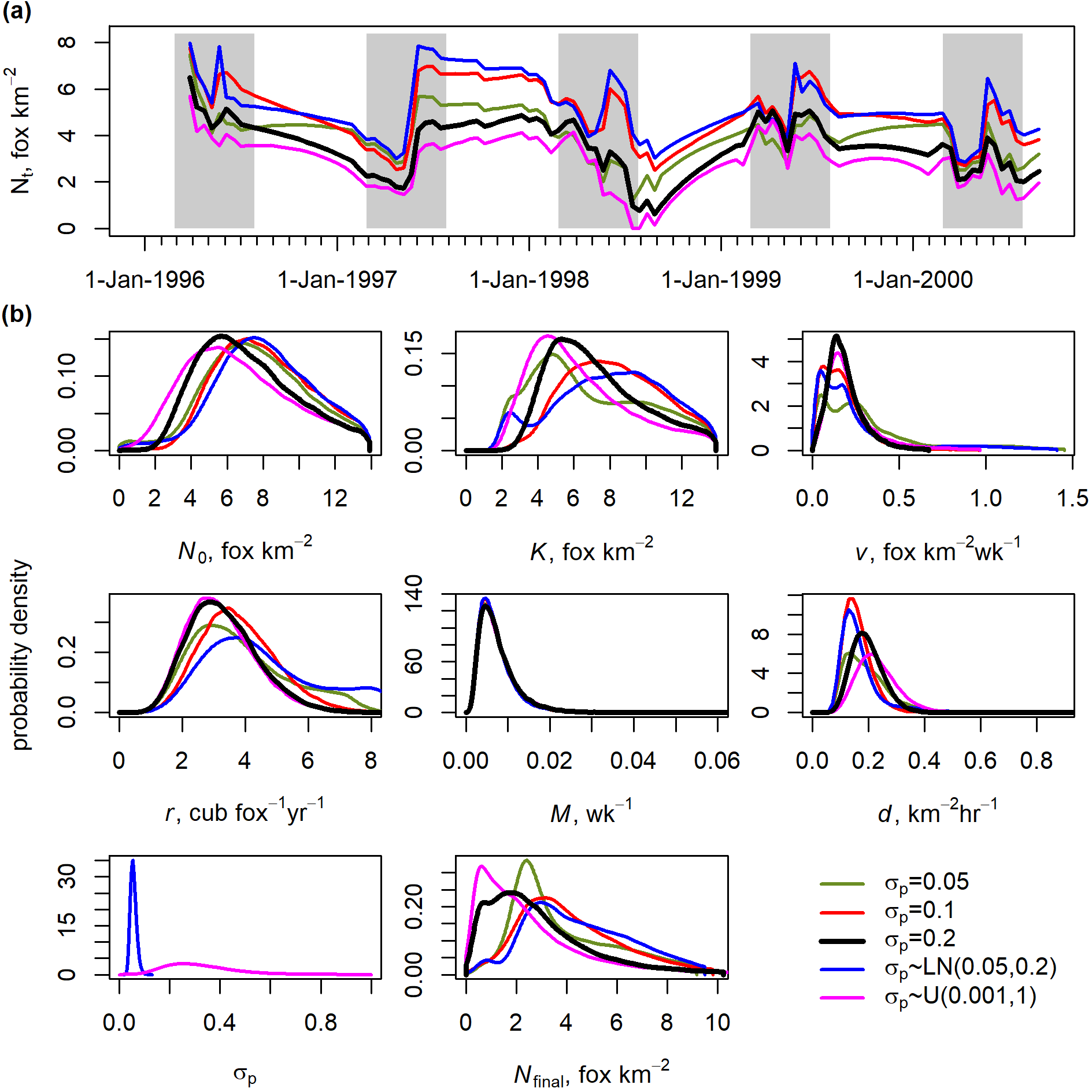

Supplement: S2 Fig — Results for DLQ showing sensitivity of a) posterior median fox density and b) marginal posterior parameter estimates to specification of the process error standard deviation, σp. The values of σp were either fixed at 0.05, 0.1, or 0.2, or were estimated using either a lognormal prior distribution with median of ln(0.05) and CV of 0.2 or a uniform prior with lower and upper bounds of 0.001 and 1.0, respectively. The reference case, where σp is fixed at 0.2, is shown in bold. (TIF) [file pone.0225201.s007.tif]

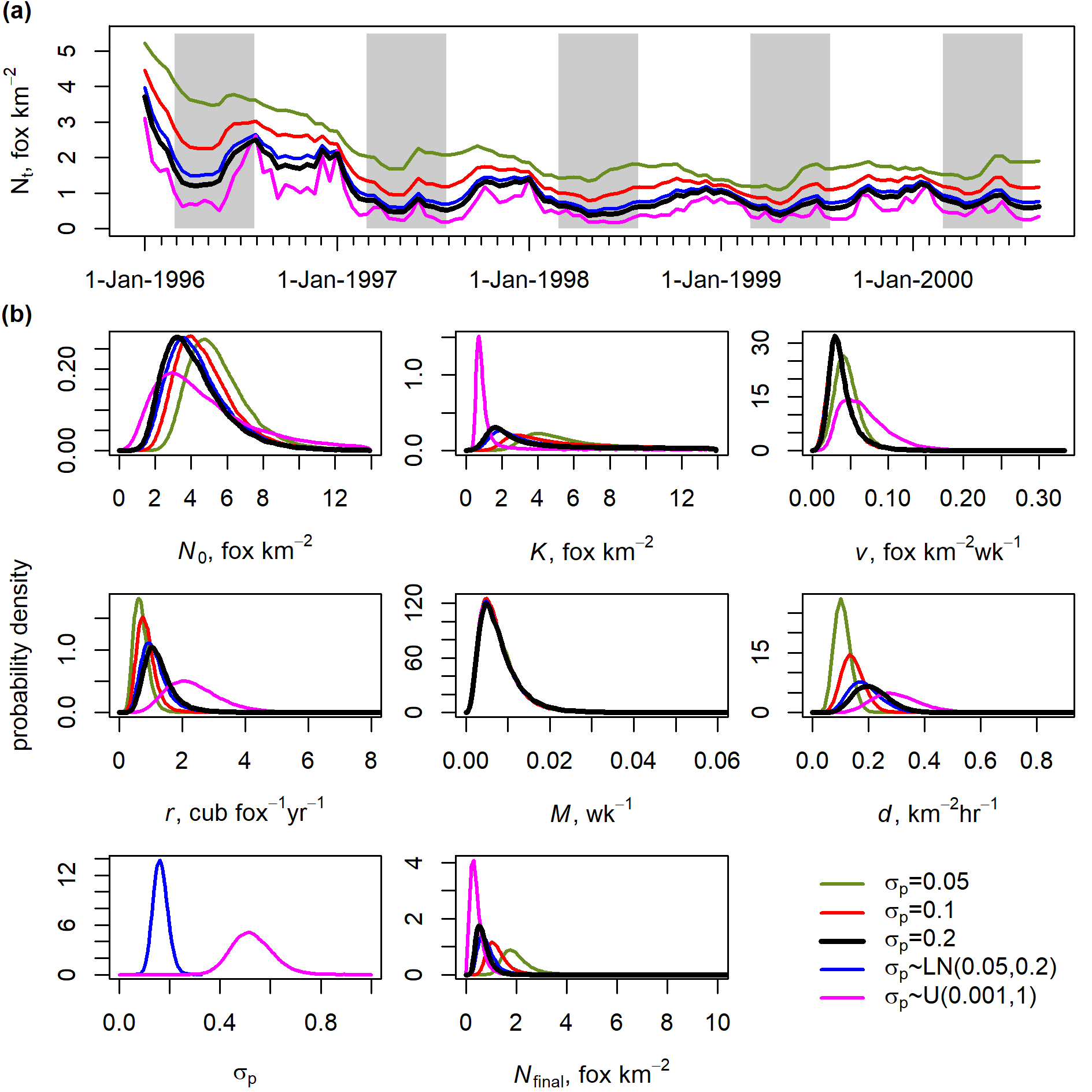

Supplement: S3 Fig — Results for VAR showing sensitivity of a) posterior median fox density and b) marginal posterior parameter estimates to specification of the process error standard deviation, σp. The values of σp were either fixed at 0.05, 0.1, or 0.2, or were estimated using either a lognormal prior distribution with median of 0.05 and CV of 0.2 or a uniform prior with lower and upper bounds of 0.001 and 1.0, respectively. The reference case, where σp is fixed at 0.2, is shown in bold. (TIF) [file pone.0225201.s008.tif]
