# Supplementary material for: Population dynamics of foxes during restricted-area culling in Britain: Advancing understanding through state-space modelling of culling records
Source: PLoS One. 2019 Nov 19;14(11):e0225201. doi: 10.1371/journal.pone.0225201 (PMC6863561; doi:10.1371/journal.pone.0225201)
Supplement: S1 Table — (PDF) [file pone.0225201.s009.pdf]

**S1 Table. Estate area, number of contributed weeks' data, number of weeks of lamping effort and lamping effort per km$^2$ in each year.**

| Estate | Area (km$^2$) | Total weeks in culling period (weeks with lamping effort) | | | | | Total lamping effort (hr km$^{-2}$) | | | | |
|---|---|---|---|---|---|---|---|---|---|---|---|
| | | 1996 | 1997 | 1998 | 1999 | 2000* | 1996 | 1997 | 1998 | 1999 | 2000* |
| BMM | 3.6 | - | - | 40 (10) | 52 (13) | 35 (12) | - | - | 7.2 | 11.2 | 9.6 |
| CHU | 3.6 | - | - | 40 (23) | 52 (31) | 35 (12) | - | - | 14.5 | 17.1 | 8.3 |
| CIP | 2.4 | 52 (23) | 52 (17) | 52 (15) | 13 (5) | - | 16.3 | 12.8 | 12.0 | 6.6 | - |
| CUL | 20.2 | 52 (26) | 52 (21) | 52 (15) | 52 (13) | 14 (3) | 5.2 | 4.0 | 2.4 | 1.8 | 0.6 |
| DLQ | 3.2 | 40 (6) | 52 (14) | 52 (13) | 52 (12) | 35 (9) | 5.1 | 13.4 | 12.2 | 11.3 | 11.3 |
| DWS | 18.2 | - | 40 (24) | 52 (39) | 36 (18) | - | - | 9.6 | 17.6 | 6.3 | - |
| EWE | 4.9 | 52 (26) | 52 (28) | 52 (29) | 52 (24) | 35 (15) | 7.8 | 7.8 | 7.5 | 6.2 | 4.2 |
| FAH | 5.4 | - | - | 40 (12) | 52 (17) | 35 (8) | - | - | 2.4 | 5.4 | 3.4 |
| FHC | 4.1 | 52 (41) | 52 (36) | 52 (37) | 52 (31) | 35 (19) | 32.0 | 29.2 | 23.3 | 29.8 | 11.0 |
| GDE | 36.4 | 52 (29) | 52 (26) | 52 (23) | 52 (21) | 35 (16) | 3.2 | 2.9 | 2.3 | 1.7 | 1.3 |
| GHT | 5.7 | - | - | 40 (38) | 52 (49) | 35 (34) | - | - | 35.7 | 49.5 | 38.1 |
| HIR | 6.1 | - | - | 40 (38) | 52 (52) | 35 (35) | - | - | 43.7 | 59.3 | 31.5 |
| HUS | 9.3 | 52 (18) | 52 (15) | 52 (26) | 52 (26) | 35 (14) | 5.8 | 4.5 | 12.0 | 11.2 | 3.6 |
| LEL | 2.4 | 52 (29) | 52 (12) | 52 (25) | 13 (6) | - | 15.7 | 5.2 | 8.3 | 1.4 | - |
| MAH | 4.9 | 52 (14) | 52 (15) | 35 (6) | - | - | 7.1 | 11.3 | 3.8 | - | - |
| NOG | 8.1 | - | 40 (29) | 52 (35) | 52 (38) | 35 (13) | - | 11.6 | 10.9 | 18.6 | 5.6 |
| NYP | 10.1 | 52 (17) | 52 (20) | 52 (37) | 35 (23) | - | 4.4 | 3.4 | 8.1 | 6.2 | - |
| OCS | 1.6 | 52 (31) | 52 (21) | 52 (28) | 13 (4) | - | 24.8 | 15.8 | 24.9 | 2.2 | - |
| RAM | 2.4 | 52 (17) | 52 (16) | 52 (28) | 52 (24) | 35 (14) | 5.3 | 9.6 | 12.0 | 9.8 | 6.4 |
| VAR | 24.3 | 52 (45) | 52 (52) | 52 (52) | 52 (51) | 35 (35) | 17.6 | 21.3 | 21.8 | 27.0 | 15.8 |
| VDL | 4.0 | 40 (22) | 52 (27) | 52 (16) | 52 (14) | 35 (10) | 18.3 | 24.8 | 17.0 | 13.7 | 9.4 |
| YZM | 4.3 | 31 (23) | 52 (25) | 52 (27) | 52 (18) | 35 (17) | 20.9 | 16.8 | 20.5 | 13.5 | 12.8 |

*max of 35 weeks (to 31 August 2000)
