# Supplementary material for: Population dynamics of foxes during restricted-area culling in Britain: Advancing understanding through state-space modelling of culling records
Source: PLoS One. 2019 Nov 19;14(11):e0225201. doi: 10.1371/journal.pone.0225201 (PMC6863561; doi:10.1371/journal.pone.0225201)
Supplement: Attachment — Submitted filename: Referee_PlosOne_jui19.pdf [file pone.0225201.s012.pdf]

REFEREE'S REPORT TO THE AUTHORS

PONE-D-19-17263
Title: Population dynamics of foxes during restricted-area culling in Britain: advancing understanding through state-space modelling of culling records

This paper addresses a very interesting topic on impact of culling on the population dynamics of foxes. I enjoyed reading this paper. I don't have many criticisms or comments

My only concern is about the length of the manuscript. It is very detailed at the expense of clarity. I am a bit lost to find the most important take home messages.
I think the authors (and editor) should consider the option of making two consecutive papers, the first one concerning modelling (including simulation-estimation analysis and sensitivity analysis) and the second one focusing on the results and consequences on population dynamics of foxes in the 22 estates.

I think a specialist of state-space modelling, who I am not, should revised the paper. However, the modelling part is well described with precious appendices. Some of the appendices (S1, S2) include modelling of certain parameters used in the model, which are very important to consider and could be added to a first paper.
Methods concerning modelling are detailed in the methods section whereas results (simulation-estimation analysis S4 appendix and sensitivity analysis) are summarized in the Results section and put in S4 appendix. On the other hand, the Results section presented essentially parameter estimations, density reconstruction and population suppression on the 22 estates, while some of the parameters are not presented in the methods section.

I would recommend publication of the manuscript, after revision.

Yours sincerely

Specific comments:

Methods
Data

Fig. 1 Please shortly explain the difference between arable a) and arable b).
Could the names of the estates, which are used in the paper, be placed on this map?

L 139-140 Lamping should also not vary too much across the 22 estates so as to considering lamping effort per km² as an index of fox culling effort.

L160-162 The authors explained their choice of a two-weekly time-step but results from a weekly time-step are also presented L346 (for simulations, S4 appendix) and in the results section.

Table 1: Add 'detectable' to fox density N_t, as mentioned L 182-183

Population process model
L195-201 Please consider summarizing this paragraph so as to clearly define $w_{t,}$ without reading S1 appendix. Something like: 'The Proportion of cubs on an estate weaned, w_t, was calculated to ensure that cubs culled at earths within each year were not removed from the model before they had been

produced. We used data from fox populations in SE England and Wales describing the probable conception dates of female foxes killed during pregnancy [2]. We fit a logistic distribution to describe the distribution of conception events in the female fox population over time and inferred a schedule of weaning.'

L206-207 The authors assumed that non culling mortality could be considered as a density-independent constant risk. This hypothesis is plausible if you consider road traffic collisions only but that's not true for diseases.

L217 K was assumed to be constant… during the period in each estate?

L218-220 Consider putting these sentences in discussion.

Observation model

Why considering d and E_t separately in Eq(6)? Does-it mean the rate of successful search in $km^2.hr^{-1}$ is supposed to be constant for an estate over years?

Prior probability distributions for parameters

L260-265 Add symbols from table 1 for clarity in immigration rate.

Table 2: add 'on a two-weekly step' in the title

MCMC simulations

L297-301 Was reparametrisation only necessarily for MCMC simulations? I think the observation equation was also rewritten when fitting to an abundance index. If so, why not putting this part in the population process model (L221-242) section ?

Culling vs. non-culling mortality

L323 Define A, area

Results

Simulation-estimation analysis

Fig. B in this appendix is not readable.

L418-419 Fig. D in S4 appendix seems to show that Fig D this is not true for K and N_0?

L421 Here, as a result, the estates that did not meet the data requirements should be given.
L422-441 It is not clear for me which estates should be excluded regarding requirements described.

Density reconstruction

L482 Replace maximum by minimum ?

Fig. 3 Fox density in the final time-step has never been defined?

L514-546 Appendices are indispensable here to understand.

Population suppression

How were pre-breeding and post-breeding fox density calculated?

Fig. 4 This figure should be revised. Maybe a histogram per estate with K, N(Feb) and N(Mar-Jul) besides would be more illustrative.

Mortality comparison
I don’t understand the interest of using cumulative mortalities (not defined in the Methods section)? How are these annual cumulative mortalities calculated?

L577-579 I don’t understand why a cumulative non-culling mortality exceeding carrying capacity after 4 years would imply a population turnover of four years in the absence of culling ?

Fig. 5 What is the scale for mortalities?

Sensitivity analyses
L 590 Why performing sensitivity analyses on six estates. This should be placed in the Methods section

I ‘m not sure Fig. 6 is very useful.

Fig. 7 Please precise a) and b)

Discussion
Parameter estimates
L677 The area YZM is also very small (4.3 km^2). Is this very high immigration rate estimated on a two-week period?
